# Sprayable biomimetic double mask with rapid autophasing and hierarchical programming for scarless wound healing

**DOI:** 10.1126/sciadv.ado9479

**Published:** 2024-08-14

**Authors:** Yuhe Yang, Di Suo, Tianpeng Xu, Shuai Zhao, Xiaoxiao Xu, Ho-Pan Bei, Kenneth Kak-yuen Wong, Qibin Li, Zijian Zheng, Bin Li, Xin Zhao

**Affiliations:** ^1^ Department of Applied Biology and Chemical Technology, The Hong Kong Polytechnic University, Hung Hom, Hong Kong SAR, China.; ^2^ Department of Biomedical Engineering, The Hong Kong Polytechnic University, Hung Hom, Hong Kong SAR, China.; ^3^ The Hong Kong Polytechnic University Shenzhen Research Institute, Shenzhen, Guangdong 518057, China.; ^4^ Department of Surgery, Li Ka Shing Faculty of Medicine, The University of Hong Kong, Hong Kong SAR, China.; ^5^ Research Center for Intelligent Aesthetic Medicine, PolyU-Hangzhou Technology and Innovation Research Institute, Hangzhou, Zhejiang 310016, China.; ^6^ Hangzhou Industrial Investment Group Co., Ltd., Hangzhou, Zhejiang, 310025, China.; ^7^ Research Institute for Intelligent Wearable Systems, The Hong Kong Polytechnic University, Hung Hom, Kowloon, Hong Kong SAR, China.; ^8^ Medical 3D Printing Center, Department of Orthopaedic Surgery, The First Affiliated Hospital of Soochow University, Suzhou, Jiangsu, China.; ^9^ Orthopedic Institute, Suzhou Medical College, Soochow University, Suzhou, Jiangsu, China.; ^10^ Research Institute for Future Food, The Hong Kong Polytechnic University, Hung Hom, Kowloon, Hong Kong SAR, China.

## Abstract

Current sprayable hydrogel masks lack the stepwise protection, cleansing, and nourishment of extensive wounds, leading to delayed healing with scarring. Here, we develop a sprayable biomimetic double wound mask (BDM) with rapid autophasing and hierarchical programming for scarless wound healing. The BDMs comprise hydrophobic poly (lactide-*co*–propylene glycol–*co*-lactide) dimethacrylate (PLD) as top layer and hydrophilic gelatin methacrylate (GelMA) hydrogel as bottom layer, enabling swift autophasing into bilayered structure. After photocrosslinking, BDMs rapidly solidify with strong interfacial bonding, robust tissue adhesion, and excellent joint adaptiveness. Upon implementation, the bottom GelMA layer could immediately release calcium ion for rapid hemostasis, while the top PLD layer could maintain a moist, breathable, and sterile environment. These traits synergistically suppress the inflammatory tumor necrosis factor–α pathway while coordinating the cyclic guanosine monophosphate/protein kinase G–Wnt/calcium ion signaling pathways to nourish angiogenesis. Collectively, our BDMs with self-regulated construction of bilayered structure could hierarchically program the healing progression with transformative potential for scarless wound healing.

## Teaser

A sprayable biomimetic double mask is constructed with hierarchical programming for scarless wound healing.

## INTRODUCTION

Extensive skin wounds resulting from burn debridement, surgical incisions, and traumatic injuries represent the most perilous forms of skin trauma and constitute a real threat to patient survival (*1*, *2*). These wounds are characterized by extensive and irregular wound areas, accompanied by severe hemorrhage and a heightened susceptibility to infection (*3*). These complications can lead to severely disrupted healing cascade such as uncontrollable hemostasis and protracted inflammation, culminating in compromised healing progression and inevitable scar formation (*4*). Clinically, various masks have been adopted for extensive wound management (*5*). Among all mask materials, hydrogels (e.g., alginate and hyaluronic acid) stand out from their counterparts (e.g., gauzes and foams) due to their maintenance of moist wound environment, absorption of tissue exudates, and biomimicry of extracellular matrix (ECM) (*6*). Recently, in situ forming sprayable hydrogels have emerged as one of the most promising solutions compared to premade hydrogels with distinct advantages including excellent portability, enhanced flexibility, and superior conformability to irregular wounds with varying dimensions (*7*, *8*). Unfortunately, the current sprayable hydrogel wound masks are unable to manage the extensive wounds with complex pathological conditions due to the lack of bleeding management, moisture and breathability modulation, and wound nourishment to support scarless wound healing. Moreover, they have insufficient tissue adhesiveness and mechanical property so they can neither provide initial stabilization of wounds nor firmly adhere and conform to body movements to protect the wound site (*9*, *10*). They are always inept at consistent maintenance of wound moisture levels due to excess water evaporation and rapid degradation (*11*). In addition, they cannot provide wounds with the necessary permeation for oxygen diffusion while preventing microbial infections (*12*). All these limitations can lead to prolonged inflammatory response, delaying skin epithelialization with scarring. Moreover, the severe vascular damage caused by extensive wounds can substantially hinder the blood supply, causing depletion of nutrients and delay in regenerative cell (e.g., fibroblasts) rescue, leading to deferred tissue regeneration (*8*).

**Fig. 1.**
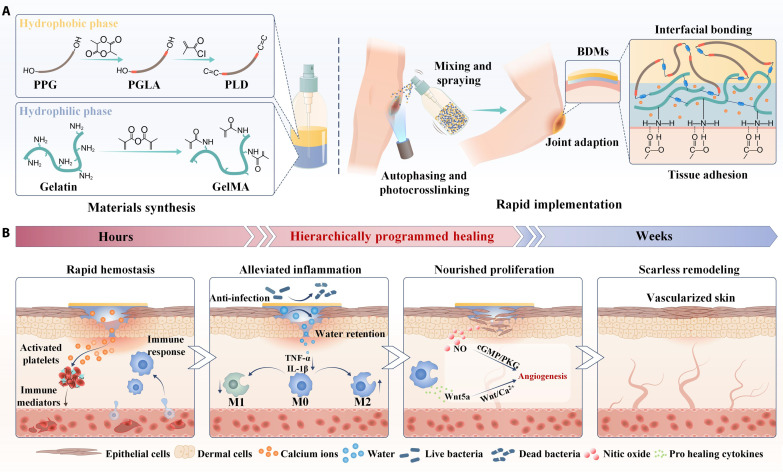
Schematics showing the sprayable BDMs with rapid autophasing and hierarchical programming for scarless wound healing. (**A**) The hydrophobic PLD and hydrophilic GelMA quickly autophase into bilayered structure after mixing and spraying, forming the BDMs after photocrosslinking with strong interfacial bonding, robust tissue adhesion, and excellent adaptation to joint movement. PPG, propylene glycol; PGLA, poly (lactide-*co*–propylene glycol–*co*-lactide). (**B**) The BDMs with stepwise hemostasis, maintenance of the moist, breathable and sterile environment, and angiogenesis nourishment can hierarchically program the dynamic healing cascade for scarless wound healing by inhibiting the inflammatory TNF-α pathway with increased M2 macrophage polarization to promote the transition from inflammatory to proliferative phenotypes, while activating and coordinating the cGMP/PKG-Wnt/Ca^2+^ signaling pathways to promote vascular reconstruction and scarless wound healing.

The natural architecture of human skin featuring a moist dermal layer beneath a horny epidermis layer serves as a concrete basis for tissue engineered masks. The protective epidermis modulates water evaporation and oxygen permeation while shielding external contaminants invasion (e.g., germs and dust) (*13*). Meanwhile, the dermis stepwisely releases coagulation factors (e.g., calcium ions) for rapid hemostasis and produce regenerative biomolecules [e.g., nitric oxide (NO)] for nourished wound healing (*14*). Inspired by structure and function of natural skin, we engineer a sprayable biomimetic double mask (BDM) with rapid autophasing and hierarchical programming for scarless wound healing (Fig. 1). The sprayable BDMs are photocrosslinkable and consist of two distinct layers: a bottom layer of hydrophilic gelatin methacryloyl (GelMA) hydrogel with calcium ion (Ca^2+^) incorporation and a top layer of hydrophobic poly (lactide-*co*–propylene glycol–*co*-lactide) dimethacrylate (PLD) polymer with triclosan (TCS) incorporation. The Ca^2+^ is an endogenous ion naturally present in the human body and plays a critical role in the blood coagulation cascade by activating various clotting factors to modulate the hemostasis process (*15*). In addition, TCS was chosen for its broad-spectrum antimicrobial properties, excellent biocompatibility, and hydrophobic nature, which facilitates its integration into the hydrophobic PLD layer (*16*). The two phases could swiftly autophase into a bilayered structure through spontaneous water/oil separation upon mixing. Compared to sequential spraying, this premixing and autophasing process not only facilitates a more user-friendly application by dispensing both materials from one single dispenser but also ensures uniform distribution across all sprayed locations to achieve consistent coverage of top PLD layer over GelMA layer across the wound. After photocrosslinking, the BDMs could be solidified within 100 s with strong interfacial bonding because of the presence of “C═C” in PLD and GelMA. The BDMs could robustly adhere to the wound site and adapt to joint movement thanks to the tissue adhesion of GelMA and elasticity of GelMA and PLD (Fig. 1A). After implementation, the BDMs could maintain a moist, breathable, and sterile microenvironment and hierarchically program the dynamic healing cascade. Initially, the soft and hydrophilic Ca^2+^-loaded GelMA could hydrate the wound and activate the coagulation cascade for rapid hemostasis. Meanwhile, the stiff and hydrophobic PLD layer encapsulating antibacterial TCS could serve as a protective epidermis to maintain the durable moist and sterile environment, while ensuring the appropriate permeability. These traits synergistically enable our BDMs to suppress the inflammatory pathway [e.g., tumor necrosis factor–α (TNF-α)] with increased M2 macrophage polarization to advance the transition of inflammation to proliferation. Afterward, the BDMs nourish angiogenesis through coactivating and coordinating the cyclic guanosine monophosphate/protein kinase G (cGMP/PKG)–Wnt/Ca^2+^ signaling pathways mediated by NO generated from GelMA’s degradation product l-arginine and Wnt5a secreted by polarized M2 macrophage to enhance the vascular reconstruction and empower scarless wound healing (Fig. 1B).

This project gives insight into the biomimicking design of the sprayable bilayer wound mask with integration of stepwise hemostasis, followed by maintenance of the moist, breathable, and sterile microenvironment and angiogenesis nourishment in succession for scarless wound healing. The project develops sprayable double mask with in situ rapid autophasing and recapitulation of natural skin’s stepwise process to facilitate healing. All components used in our double mask composed exclusively of endogenous elements inherent to the human body without living cells or tissues, thus circumventing potential ethical obstacles for clinical application. These traits and practicalities are poised to garner interest from hospitals and enterprises seeking to translate our findings. Collectively, this research offers a highly translational approach with substantial benefits for patients with extensive wounds, alleviating burdens on families and society.

## RESULTS

### Preparation and characterization of GelMA and PLD base materials

Our sprayable BDMs were composed of hydrophilic GelMA (denoted as G) and the hydrophobic PLDs (denoted as P*m*L*n*D; *m* and *n* refer to unit length of propylene glycol and lactide). To optimize the materials formulations used in our BDMs, we firstly synthesized a series of GelMA with 30, 60, and 90% C═C modification degree (i.e., G30, G60, and G90) and PLD with different molecular weights (i.e., P7L2D, P7L4D, P17L4D, and P34L4D), respectively, following our previous protocol (*17*, *18*). The chemical structures of different GelMA and P*m*L*n*D formulations were characterized using Fourier transform infrared (FTIR) spectroscopy and the ^1^H nuclear magnetic resonance (^1^H NMR) spectrum (fig. S1). These analyses confirmed the successful synthesis of the two materials. Notably, all formations of GelMA and P*m*L*n*D contained abundant C═C moieties in their chemical structures, enabling the cophotocrosslinking process.

Then, we prepared the working solution of GelMA (10%, w/v, in water) and P*m*L*n*D and performed the rheology evaluation to validate the sprayability of the two phases (fig. S2, A and B). We found that all the GelMA solution (i.e., G30, G60, and G90) presented shear-thinning behavior with excellent fluidity, which is crucial for the spraying process. Regarding the P*m*L*n*D polymers, the P7L2D demonstrated the lowest viscosity (78 mPa·s) due to shortest molecular chain length, making it particularly suitable for spraying applications. In contrast, other P*m*L*n*D formulations (i.e., P7L4D, P17L4D, and P34L4D) exhibited substantially higher viscosities (>900 mPa·s), posing challenges for their use in spray-based delivery systems. In addition, we evaluated the tensile mechanical properties of different GelMA and P*m*L*n*D formulations after cross-linking (fig. S2, C and D). All GelMA formulations exhibited remarkable elasticity with over 60% strains, with G90 showing the highest tensile modulus (~85 kPa), which is comparable to the natural dermis layer (*19*). For the P*m*L*n*D formulations, P7L2D displayed over 65% strain and a tensile modulus of ~13 MPa, which is similar to the natural epidermis, enabling the protection of the bottom hydrogel layer (*20*). Furthermore, we evaluated the degradation rate of different GelMAs and P*m*L*n*Ds using collagenase (CL) solution (0.5 U/ml) and deionized (DI) water, respectively (fig. S2, E and F). As anticipated, G30 and G60 with loose cross-linking network exhibited rapid enzymatic degradation within 5 days, while the G90 groups displayed slower degradation for 14 days due to the tighter cross-linking network. The longer degradation time is beneficial for healing of extensive wounds. Compared to GelMA, all P*m*L*n*D formulations demonstrated much slower degradation rates, degrading ~15% in 28 days. This enables the long-term protection of the hydrogel bottom layer and the wound bed. We additionally tested the photocrosslinking kinetics of G90 and P7L2D via real-time storage modulus monitoring (fig. S3, A and B). Upon blue light (405 nm) irradiation, the storage modulus of G90 and P7L2D started increasing instantly and plateaued after around 90 s, indicating the completion of photocrosslinking reaction. This rapid photocrosslinking kinetics observed in G90 and P7L2D with instant storage modulus increase enables the materials’ transition from liquid to solid state in a very short period, which provides instant protection of wound bed.

Given these findings including sprayability, mechanical properties, degradability, and photocrosslinkablity, G90 and P7L2D were selected as the optimized formulations for the bottom and upper layers of our BDM. For the simplicity, G90 and P7L2D were denoted as GelMA and PLD in the subsequent studies, respectively.

### Fabrication and characterization of the sprayable BDMs

**Fig. 2.**
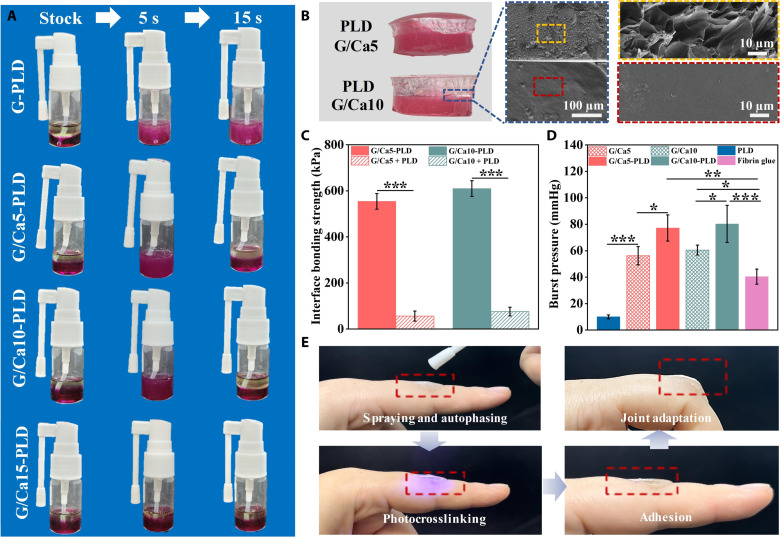
Formulation and implementation evaluation of the sprayable BDMs. (**A**) The autophasing capacity evaluation of GelMA (G) and PLD solution in stock status and 5 and 15 s after mixing. The G/Ca5, G/Ca10, and G/Ca15 denoted GelMA with 5, 10, and 15% CaCl_2_ incorporation, respectively. (**B**) The macroscopic and SEM images of the formed BDMs after photocrosslinking. (**C**) The quantification of the interfacial bonding strength of the two layers evaluated by the lap shear test. (**D**) Burst release pressure of different materials for the evaluation of the tissue adhesion. (**E**) Demonstration of rapid implementation of the sprayable BDMs with strong tissue adhesion and the joint movement adaptation. Sample size *n* = 3 for all experiments by a one-way or two-way analysis of variance (ANOVA) with a Tukey’s post hoc test for multiple comparisons. Data are presented as means ± SD. *P < 0.05, **P < 0.01, and ***P < 0.001 are considered statistically significant.

After base material optimization, we formulated the sprayable autophasable BDM through the natural water/oil separation process between the optimized hydrophilic GelMA (water phase) and the hydrophobic PLD (oil phase). Although the pristine GelMA and PLD (G-PLD) could form distinct two layers in stock status, they formed emulsion after mixing and could not resume the bilayered structure after 15 s (Fig. 2A). To this end, we incorporated CaCl_2_ into GelMA as a coagulant to facilitate the water/oil separation. Ca^2+^ has proven ability to reduce surface tension of droplets in emulsion to allow ready phase separation (*21*, *22*). As expected, we found that the GelMA with 5 wt % of CaCl_2_ (G/Ca5) and 10 wt % of CaCl_2_ (G/Ca10) could maintain the homogeneous mixture state for 5 s after thorough mixing and spontaneously resume the bilayered structure within 15 s (Fig. 2A and movie S1). In contrast, the GelMA with 15 wt % of CaCl_2_ (G/Ca15) exhibited immediate restoration of the bilayered structure (<5 s) after mixing. This superfast autophasing rate proved to be less practical and could potentially occur even before the spraying process is completed, complicating the application and potentially leading to failure of double-layer formation. Therefore, we selected G/Ca5 and G/Ca10 with moderate autophasing rate for further studies to ensure the mixture spraying and rapid water/oil separation after deployment. We then used blue light (405 nm, 5 mW/cm^2^) to cross-link the autophased spray to construct the BDMs. We found that both the G/Ca5-PLD and G/Ca10-PLD groups presented distinctive bilayered structure within 100 s (Fig. 2B and movie S2). To further elaborate the necessity and efficiency of our system design over the sequential spraying, we compared the constructed double mask with the different preparation methods. We found that our spraying and autophasing process could ensure both materials to be evenly deposited in all sprayed location to achieve consistent and uniform coverage of top PLD layer over GelMA layer across the wound for effective wound protection from external contaminants while maintaining a moist environment. However, this complete and uniform coverage was difficult to achieve with sequential spraying process due to the requirement of precise alignment during each application step (fig. S4). To observe the microstructure of the formed BDMs, we performed the scanning electron microscope (SEM) evaluation and found that the G/Ca10 layer presented porous microstructure, while the PLD layer presented dense microstructure. In addition, we found that the G/Ca10 could form seamless interface with PLD after cross-linking, indicating the tight bonding between the two phases (Fig. 2B).

During the clinical practice, it is imperative for wound masks to effectively adhere to the natural tissue and accommodate to the complex joint movement without layer delamination, which underscores the need for the strong interfacial bonding, robust tissue adhesion, and remarkable elasticity. We thus performed the mechanical shear test to quantify the interfacial bonding strength of BDMs after co–cross-linking (Fig. 2C). The separately cross-linked G/Ca5, G/Ca10 and PLD samples (denoted as G/Ca5 + PLD and G/Ca10 + PLD) were used as control. We found that both the G/Ca5-PLD and G/Ca10-PLD groups exhibited substantially stronger interfacial bonding strength (~534 kPa for G/Ca5-PLD and ~576 kPa for G/Ca10-PLD) than the control groups (~37 kPa for G/Ca5 + PLD and ~72 kPa for G/Ca10 + PLD) due to the covalent bond formation between the GelMA and PLD (Fig. 2C). We further prepared the standard tensile bar by the G/Ca5 and PLD, which could bear the bending process and crack at G/Ca5 side under the tensile testing, further demonstrating the strong interfacial bonding (fig. S5, A and B). We additionally adopted the standard burst release test to evaluate the adhesive property of our BDMs and used commercial fibrin glue as a control. We found that the G/Ca5 and G/Ca10 groups presented strong tissue adhesion due to the formation of hydrogen bonds between GelMA and tissue and covalent bonds between the methacryloyl groups of GelMA and the amine groups of tissue during photocrosslinking (Fig. 2D) (*23*). Notably, we found that the G/Ca5-PLD and the G/Ca10-PLD groups exhibited further increased burst pressure compared to commercially available fibrin glue, with approximately 1.91- and 1.99-fold enhancement, which might be attributed to the increased stiffness of the double-layer structure indicating the robust tissue adhesion of our BDMs. In addition, to provide a deeper insight into the adhesion properties of our BDMs, we performed the lap shear tests and found that our BDMs showed strong adhesion strength (over 125 kPa) to the natural tissue in the presence of shear forces (fig. S6). These results further confirmed the excellent adhesion capacity of our BDMs. Furthermore, we performed tensile test to characterize the mechanical properties of the BDMs (fig. S7). We found that the BDMs also presented superior elasticity and could maintain the seamless interface during deformation. Then, to evaluate the adaptiveness of our BDMs to irregular wounds, we used various molds such as moon, star, and triangle shapes to represent the irregular wound geometries. We observed that the BDMs showed excellent conformability to different irregular molds with the formation of bilayered structure (fig. S8). To validate the stability of our BDMs against external mechanical interferences (e.g., scratches), we evaluated resilience of our BDMs to external mechanical scratches (fig. S9). We found that G/Ca5 with sole hydrogel presented susceptibility to visible scratches, highlighting the fragile nature of conventional hydrogels. In contrast, our BDMs exhibited negligible scratch marks, demonstrating the excellent protective effect of the PLD layer. These results demonstrate the excellent stability of our BDMs in resisting external mechanical interference (e.g., scratches).

Last, we validated the rapid implementation capacity of BDMs simulating practical situation. After spraying, autophasing, and photocrosslinking, we found that the BDMs could be swiftly constructed with robust adhesion to the hand joint and excellent movement adaptation (Fig. 2E). Moreover, we validated the adaptability of our BDMs on different skins with varied mechanical properties and dryness simulating practical situations. We found that the BDMs could conform to different contours and maintain integrity to the elbow, knee, and heel, further demonstrating that our BDMs can provide effective treatment across a broad range of clinical scenarios (fig. S10).

### Creation of rapid hemostasis using the autophasable BDMs

**Fig. 3.**
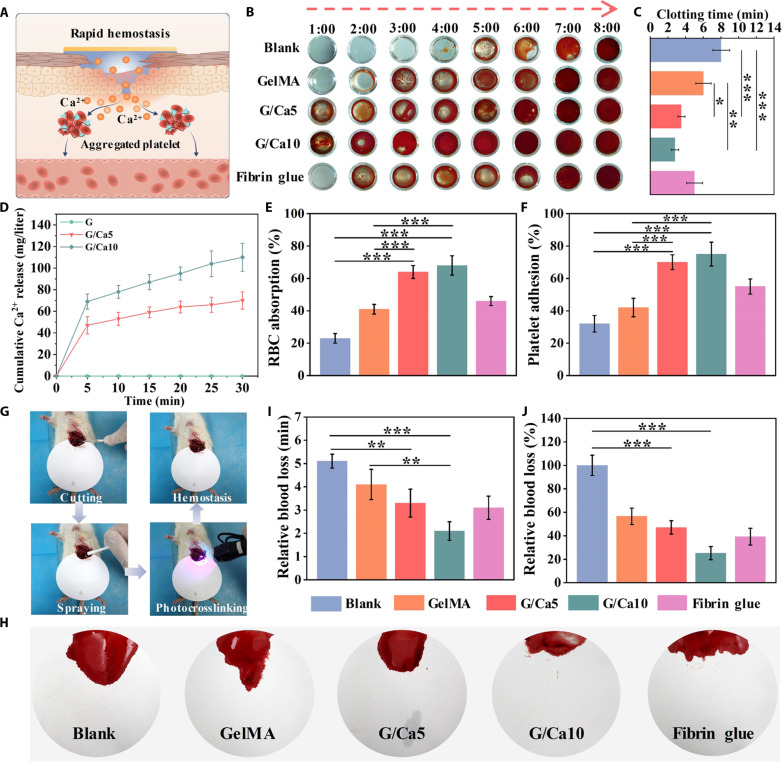
Creation of rapid hemostasis using the autophasable BDMs. (**A**) Schematic showing the autophased BDMs with calcium ion release to modulate the coagulation cascade. (**B**) Whole blood clotting assay with clot formation and (**C**) clotting time quantification for GelMA, G/Ca5, G/Ca10, and fibrin glue. (**D**) Quantification of Ca^2+^ release in G/Ca5 and G/Ca10 samples. Quantification of (**E**) RBCs and (**F**) platelet adhesion of different samples. (**G**) Schematic illustration showing the typical experimental procedure of the rat liver injury model. (**H**) Bloodstain images with the quantification of (**I**) hemostasis time and (**J**) relative blood loss for different samples. The G/Ca5 and G/Ca10 denoted GelMA with 5 and 10% CaCl_2_ incorporation, respectively. Sample size *n* = 3 for all experiments by a one-way ANOVA with a Tukey’s post hoc test for multiple comparisons. Data are presented as means ± SD. *P < 0.05, **P < 0.01, and ***P < 0.001 are considered statistically significant.

After injury, the natural stepwise wound healing cascade initiates immediately starting with hemostasis. Clinically, uncontrollable bleeding could impair the organized healing cascade with overactivated immune response (*15*). Thus, we evaluated the effect of Ca^2+^ loaded in the bottom GelMA layer on the hemostasis performance of the autophasable BDMs and used the naked PLD as the upper layer without the TCS incorporation (Fig. 3A). We first performed the in vitro whole blood clotting assay with commercial fibrin glue used as control (Fig. 3B). We found that the GelMA and fibrin glue presented reduced blood clotting time compared to blank group due to the activated platelet aggregation (*24*). Noticeably, the G/Ca5 and G/Ca10 groups demonstrated markedly accelerated blood clotting time with ~3.8 and ~3.2 min, respectively, which could be attributed to the massive release of Ca^2+^ from the BDMs (Fig. 3C). We found that Ca^2+^ in both groups could be burst-released within 5 min and attributed to the hydrophilic nature and loose polymeric network of GelMA (Fig. 3D). This steep Ca^2+^ release kinetics was decisive for primary coagulation through regulating hemagglutination and platelet aggregation. We further evaluated the attachment of red blood cells (RBCs) and platelets on the materials. We found that the G/Ca5 and G/Ca10 groups exhibited ~2.8- and ~3.0-fold increase in RBC absorption compared with the blank group (Fig. 3E). Moreover, the G/Ca5 and G/Ca10 groups also showed ~2.2- and ~2.3-fold increase in platelet adhesion compared with the blank group (Fig. 3F). To further validate the Ca^2+^-modulated hemostasis of our BDMs, we prepared thrombin-free fibrin glue cross-linked by calcium chloride with the same Ca^2+^ concentration (i.e., 10 wt %) as the G/Ca10 BDM (denoted as fibrin/Ca10) (fig. S11A) (*25*). We found that this thrombin-free fibrin glue presented comparable in vitro hemostasis capacity in terms of the blood clotting time, RBC absorption, and the platelet adhesion (fig. S11, B to D). These findings not only underscored the excellent hemostatic capacity of our BDMs but also highlighted the critical role of Ca^2+^ in modulating rapid hemostasis. However, the preparation time required for this thrombin-free fibrin glue to achieve full cross-linking exceeded 12 hours, rendering it less practical for in vivo studies. Consequently, we continued using commercially available fibrin glue in subsequent experiments.

To give more insight of the excellent hemostasis performance of the BDMs, we further examined the in vivo hemostatic performance using the rat liver injury model to simulate scenarios with parenchymal bleeding that can be challenging because the tissue may not readily contract to close off small blood vessels (*26*). Following the liver incision, various BDMs were applied and photocrosslinked at the injury site (Fig. 3G). We found that the BDM was not disturbed despite the presence of excessive blood loss and could effectively form the wound mask at the site of application, adhering well to the tissue. This could be attributed to the instant storage modulus increase after photocrosslinking and the excellent tissue adhesion capacity of our BDMs. The hemostasis performance of different groups was evaluated by the hemostatic time and the bloodstains on the filter paper (Fig. 3H). We found that the G/Ca10 group achieved complete hemostasis in ~2.1 min, while the control and fibrin glue groups took around 5 and 3.1 min (Fig. 3I). The G/Ca10 group also presented substantially reduced blood loss compared to other groups (Fig. 3J). Moreover, we adopted the rat tail amputation model to mimic traumatic or emergency scenarios, such as first aid situations and battlefield injuries, where quick and efficient hemostasis is essential (fig. S12, A and B) (*24*). Immediately after tail cutting, different materials were deployed to the corresponding wound sites, followed by light irradiation to cross-link the materials. We found that the G/Ca5 and G/Ca10 groups could effectively combat this instant bleeding and the G/Ca10 group showed minimum blood loss and the shortest hemostasis time (~1.2 min), which is 1.97- and 1.92-fold less than the fibrin glue (fig. S12, C and D). To conclude, our developed autophasable BDMs exhibited remarkable rapid hemostatic performance, with the G/Ca10 group demonstrating superior potential for rapid hemostasis.

### Maintenance of moist, breathable, and sterile environment using the autophasable BDMs

**Fig. 4.**
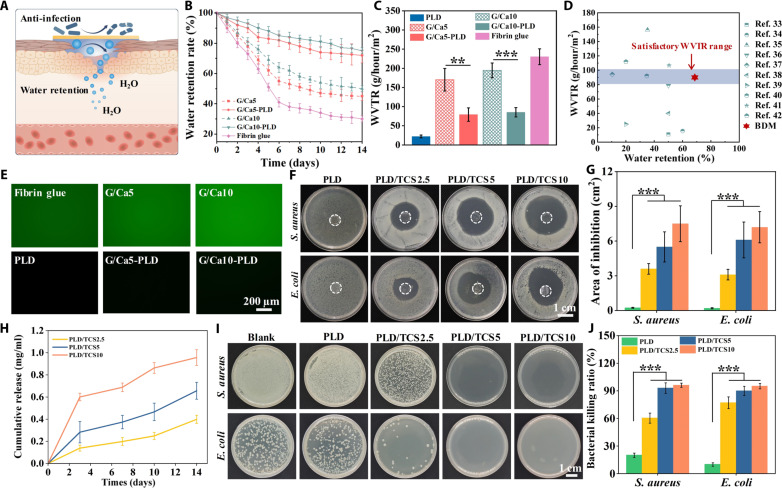
Maintenance of moist, permeable, and sterile environment using the autophasable BDMs. (**A**) Schematic showing the autophasable BDMs with PLD layer for the maintenance of moist, permeable, and sterile environment. (**B**) Water retention rate and (**C**) WVTR of different samples. (**D**) Comparison of water retention rate on day 7 or equilibrium state and WVTR with existing wound dressings. (**E**) Fluorescence images of the adsorbed BSA-FITC protein on different samples. (**F**) Representative images of inhibition zone after 24 hours of incubation of *E. coli* and *S. aureus* with PLD, PLD/TCS2.5, PLD/TCS5, and PLD/TCS10 samples. (**G**) Quantitative analysis of the inhibition zone. (**H**) Cumulative release of TCS from PLD/TCS2.5, PLD/TCS5, and PLD/TCS10 samples for 14 days. (**I**) Representative images of Luria-Bertani (LB) plates after 24 hours of incubation of *S. aureus* and *E. coli* with different samples after 14 days of degradation. (**J**) Quantitative analysis of the in vitro antibacterial efficiency. Sample size *n* = 3 for all experiments by a one-way or two-way ANOVA with a Tukey’s post hoc test for multiple comparisons. Data are presented as means ± SD. **P < 0.01 and ***P < 0.001 are considered statistically significant.

In our BDMs, we hypothesize the upper PLD layer functions as a biomimetic epidermis to effectively maintain the moist, breathable, and sterile environment (Fig. 4A). The moist, breathable, and sterile environment has been widely recognized as key elements for natural wound healing (*27*). To this end, we started with evaluation of the long-lasting moist maintenance capacity of our BDMs by quantifying the water retention rate when exposed to the air (Fig. 4B). We found that the fibrin glue groups lose the water rapidly due to the uncontrollable water evaporation. The G/Ca5 and G/Ca10 groups presented increased water retention. This could be attributed to the excellent water absorption capacity of CaCl_2_ (*28*). Notably, the BDMs presented durable water retention capacity with over 70% water retention after 14 days due to the presence of the upper hydrophobic PLD layer and the CaCl_2_ incorporation that could substantially reduce the water volatilization. This long-lasting moist maintenance could ensure the durable hydration of the wound during regeneration. In addition to the water retention, the suitable breathability of wound masks is also important for effective oxygen and waste diffusion. Thus, we first evaluated the controlled permeable capacity of our BDMs through quantification of the water vapor transmission rate (WVTR) (Fig. 4C). We found that the naked hydrogel groups (i.e., G/Ca5, G/Ca10, and fibrin glue) showed WVTR (over ~170 g/hour per square meter), which may cause fast wound dehydration due to porous structure of the hydrogels. Oppositely, the PLD group with dense microstructure and high hydrophobicity presented markedly decreased permeability with WVTR (~33 g/hour per square meter), which may cause infection with delayed wound healing (*29*). Encouragingly, we observed that the BDMs (e.g., G/Ca5-PLD and G/Ca10-PLD) exhibited moderate WVTR of ~80 and ~85 g/hour per square meter, respectively, which could be attributed to the unique bilayered structure. This WVTR value was close to the satisfactory WVTR range (~80 to 100 g/hour per square meter) for wound dressings (*30*, *31*). In addition to water vapor transmission, the adequate oxygen supply also plays a critical role in promoting wound healing by facilitating various cellular processes such as collagen synthesis and angiogenesis. Thus, we also quantified the oxygen permeability of our BDMs. We found that all BDMs formulations exhibited sufficient oxygen permeability (>7 mg/ml) (fig. S13), ensuring the adequate oxygen supply, which is crucial for enhanced wound healing processes (*32*). These results confirmed that our BDMs could effectively maintain appropriate permeability for water and oxygen diffusion to enhance wound regeneration. Compared with existing wound dressings, our BDMs could realize an excellent balance between the long-lasting moisture retention and proper permeability (WVTR) for wound management, which is the critical for effective wound healing (Fig. 4D) (*33*–*42*).

In addition, the wound dressings are also susceptible to biofouling and bacterial infection after implementation, leading to a deferred healing process and scar formation. Thus, we characterized the antifouling performance of our BDMs through the protein absorption evaluation using fluorescein isothiocyanate–labeled bovine serum albumin (FITC-BSA) as a model foulant. We found that the hydrophilic G/Ca5, G/Ca10, and fibrin glue groups presented obvious fluorescence signals indicating substantial protein adsorption (Fig. 4E and fig. S14). Nevertheless, the BDMs (e.g., G/Ca5-PLD and G/Ca10-PLD) with dense and hydrophobic PLD toppings showed neglectable fluorescence, indicating the BDMs’ excellent antifouling capacity. To further ensure the elimination of the possible infection, we incorporated TCS into the PLD layer. We used TCS due to the following reasons: (i) It is a broad-spectrum antimicrobial agent with excellent biocompatibility (*16*); (ii) it is hydrophobic, allowing for easy incorporation into the hydrophobic PLD. To validate the biocompatibility of PLD with different concentrations of TCS (e.g., 2.5, 5, and 10 wt %, denoted as PLD/TCS2.5, PLD/TCS5, and PLD/TCS10), we cultured the NIH/3T3 fibroblasts with the extraction medium of different samples. In the Live/Dead assay, the PLD/TCS2.5 and PLD/TCS5 groups presented over 95% cell viability, while the cell viability of the PLD/TCS10 group decreased to less than 80% (fig. S15, A and B). The cell proliferation further confirmed that the PLD/TCS2.5 and PLD/TCS5 groups presented no detrimental effect on cell growth, while the PLD/TCS10 group showed compromised biocompatibility (fig. S15C). Next, we evaluated the bactericidal capacity of different samples by disc diffusion method of the *Escherichia coli* (Gram negative) and *Staphylococcus aureus* (Gram positive). After contact for 24 hours, we found that the PLD with TCS incorporation exhibited obvious inhibition zones for both bacteria compared to the naked PLD group (Fig. 4F). The PLD/TCS5 and PLD/TCS10 groups showed significantly larger inhibition area compared to PLD/TCS2.5 group (Fig. 4G). Note that our PLD exhibited sustained TCS release for 14 days, which is critical for the effective bacterial inhibition throughout the wound recovery cycle (Fig. 4H). Thus, we used the conditioned samples after 14 days for the spread plate method to evaluate the long-term antibacterial activity. We found that the PLD/TCS5 and PLD/TCS10 groups still presented almost 100% antibacterial capacity, confirming its long-term antimicrobial properties (Fig. 4, I and J). In all, our developed BDMs, featuring the protective PLD layer acting as epidermis, could effectively maintain the moist, permeable, and sterile environment to facilitate wound healing.

### Nourishment of angiogenesis using the autophasable BDMs

**Fig. 5.**
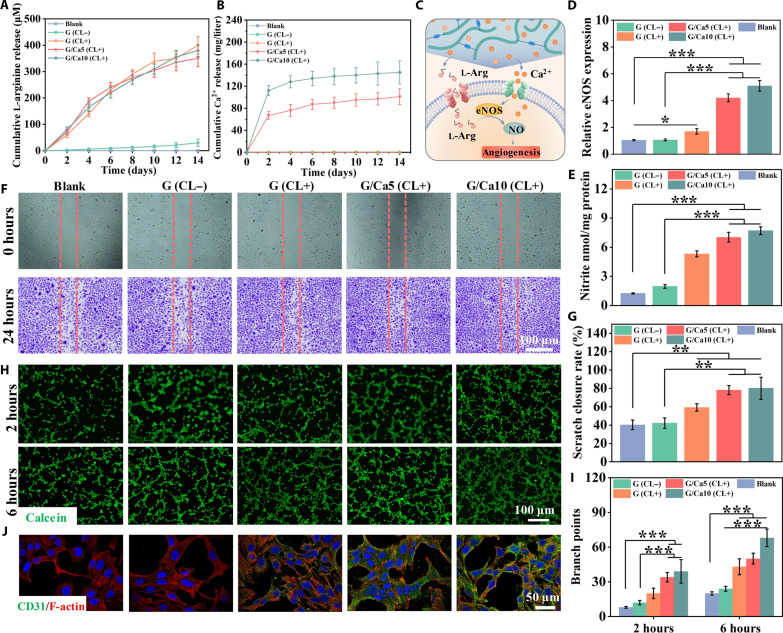
Angiogenesis nourishment using the sprayable BDMs. Release profile of (**A**) l-arginine and (**B**) Ca^2+^ from different samples CL+ or without CL (CL−). (**C**) Schematic showing the synergistically nourishing effect of the released Ca^2+^ and l-arginine with NO production. (**D**) Relative eNOS expressions and (**E**) NO generation in HUVECs cultured with different conditioned media. (**F**) Representative images of the scratch assay treated with different conditioned media before and after 24 hours. (**G**) Quantitative analysis of scratch closure area (%). (**H**) The endothelial tube formation of HUVECs after 2 and 6 hours. (**I**) Quantification of branch points of the newly formed tube. (**J**) CD31 immunofluorescence staining of HUVECs cultured with different conditioned media after 3 days of incubation. Sample size *n* = 3 for all experiments by a one-way or two-way ANOVA with a Tukey’s post hoc test for multiple comparisons. Data are presented as means ± SD. **P < 0.01 and ***P < 0.001 are considered statistically significant.

After the rapid hemostasis and protective PLD layer formation, the wound healing process continues underneath the PLD layer. Notably, the vascular damage in severe wounds can exhaust the blood circulation to hinder the proliferation stage (*43*). That is, it is desirable for our BDMs to promote angiogenesis during wound healing. In this study, we prepared BDMs with G/Ca5 and G/Ca10 as bottom layer and naked PLD as the upper layer. We used the human umbilical vein endothelial cells (HUVECs) as the model cell to assess angiogenesis and used the CL to simulate the matrix metalloproteinase (MMP)–rich wound bed. First, we quantified the release of the Ca^2+^ and l-arginine from GelMA in the presence of CL (Fig. 5, A and B). We found that all groups with CL (CL+) could sustain the release of l-arginine for 14 days (Fig. 5A). Notably, the G/Ca5 and G/Ca10 groups exhibited sustained release of residual Ca^2+^ for 14 days after the initial burst release in hemostasis stage (Fig. 5B*)*. Moreover, the G/Ca10 group showed higher Ca^2+^ release than that of G/Ca5. This copresence of the l-arginine and Ca^2+^ is expected to nourish the angiogenesis by facilitating NO synthesis (Fig. 5C) (*17*).

We further prepared the conditioned medium following the International Organization for Standardization (ISO) 10993 standard to further characterize the nourished angiogenesis (*44*). In Live/Dead staining, we found that most cells were alive in all groups and the G/Ca10 (CL+) group presented the highest cell proliferation rate, indicating the excellent biocompatibility of the conditioned medium (fig. S16, A to C). Then, we measured the level of endothelial nitric oxide synthase (eNOS) expression in HUVECs with different conditioned media. We found that the cells in G/Ca5 and G/Ca10 groups with Ca^2+^ release showed significantly elevated eNOS expression, suggesting that the Ca^2+^ could increase the eNOS expression in a dose-dependent manner (Fig. 5D). Consequently, we measured the NO production of the HUVECs and found that those cultured with released l-arginine and Ca^2+^ could generate more NO (Fig. 5E). This NO production was also confirmed by the 4-amino-5-methylamino-2′,7′-difluorofluorescein diacetate (DAF-FM DA) fluorescence NO probe staining and quantification, indicating the synergetic promotion effect of the l-arginine and Ca^2+^ in NO production (fig. S17, A and B).

Given the well-established role of NO in mediating angiogenesis during wound healing, we evaluated the angiogenic potential of HUVECs using conditioned culture medium. To assess cell migration, a critical physiological process in wound healing, we used cell scratch assay (Fig. 5, F and G). We found that the G/Ca5 (CL+) and G/Ca10 (CL+) demonstrated significantly faster migration and wound closure rate after 24 hours of incubation, confirming the capability of NO for enhancing cell migration. We further conducted the tube formation assay to determine the effect of NO on in vitro vascularization of HUVECs (Fig. 5, H and I). We observed more tubular structures formed in G/Ca5 (CL+) and G/Ca10 (CL+) after 6 hours of incubation (Fig. 5H). In particular, the G/Ca10 (CL+) group presented ~3.2-fold increased branching points compared to the blank group, indicating its exceptional potential to promote vascularization (Fig. 5I). In addition, platelet endothelial cell adhesion molecule (CD31), a comprehensive maker of angiogenesis, was found to express a similar tendency (Fig. 5J and fig. S18). Together, these results supported the notion that BDMs could be degraded in wound site, associated with released l-arginine and Ca^2+^ to generate NO to nourish the angiogenesis.

### BDMs empowered scarless wound healing in full-thickness rat skin wound model

**Fig. 6.**
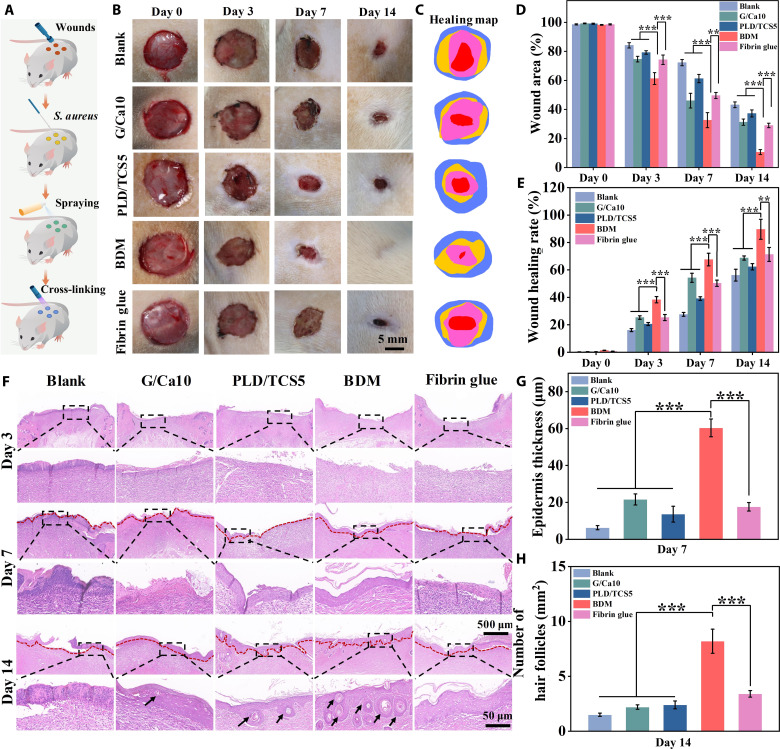
BDMs empowered scarless wound healing in full-thickness rat skin wound model. (**A**) Schematic illustration showing the construction of *S. aureus*–infected full-thickness skin defect model and the implementation of different sprayable masks. (**B**) Representative wound images and (**C**) the wound healing map treated with different wound masks. Quantification of (**D**) wound closure rate and (**E**) wound healing rate of different wound masks. (**F**) H&E staining of wound sections after 3, 7, and 14 days of treatment. The second row was the magnified image of the marked area. The red dashed lines indicate the boundary of epithelium, and the black arrows indicate the hair follicles. Quantification of (**G**) epidermis thickness and (**H**) number of hair follicles in H&E staining. Sample size *n* = 3 for all experiments by a one-way or two-way ANOVA with a Tukey’s post hoc test for multiple comparisons. Data are presented as means ± SD. **P < 0.01 and ***P < 0.001 are considered statistically significant.

The previous results demonstrated that our BDMs with PLD/TCS5 and G/Ca10 layers presented excellent performance in maintaining a moist, breathable, and sterile wound environment, rapid hemostasis, and nourishment of angiogenesis. Thus, we prepared the BDM with this formulation for further studies. As biocompatibility is crucial for the therapeutic application of wound masks, we evaluated the biocompatibility of our BDM (i.e., PLD/TCS5 + G/Ca10) in accordance with the ISO 10993 standard using NIH/3T3 cells. Bright-field and fluorescent Live/Dead staining images revealed regular cell morphology and scarce cell death in the BDM group, with viability exceeding 95% at all time points (fig. S19, A and B). Furthermore, the BDM group exhibited a proliferation trend comparable to that of the blank and fibrin glue groups (fig. S19C). These findings underscore the excellent biocompatibility of our BDMs. As the presence of TCS in the final BDM may change the previous hemostatic outcome compared to the fibrin glue without antibacterial agent due to the natural antimicrobial properties of activated platelets (*45*), we first evaluated the hemostasis performance of the optimized BDM with TCS incorporation and used fibrin glue as a control with rat liver injury model (fig. S20). We found that the BDM with TCS incorporation (G/Ca10-PLD/TCS10) presented comparable hemostasis performance with the BDM without TCS incorporation (G/Ca10-PLD) and both were superior to fibrin glue in terms of whole blood clotting and in vivo hemostatic efficacy, indicating the robustness of hemostasis performance of our BDMs. Then, we evaluated the therapeutic wound healing efficacy of the BDM using an *S. aureus*–infected full-thickness rat skin wound model (Fig. 6A). The PLD/TCS5 and G/Ca10 single-layer masks and the commercial fibrin glue were used as control.

After the establishment of the animal model and implementation of different masks, we observed the dynamic wound healing process by macroscopic images (Fig. 6B). We found that the BDM group presented smaller wound area than other groups. Contrarily, the G/Ca10 and fibrin glue groups suffered from bacterial infection after 7 days of treatment with yellow exudate and pus covering the wound (Fig. 6B). The healing map revealed that the BDM group had almost completely healed with less than 10% of the initial wound area after 14 days, while the other groups presented obvious incomplete healing sections (Fig. 6C). In addition, the wound area and wound healing rate quantification further confirmed the significantly accelerated wound healing process with the BDM (Fig. 6, D and E). Then, hematoxylin and eosin (H&E) staining of the skin tissue was applied to evaluate the dynamic wound healing progression. On day 3, the blank and G/Ca10 groups exhibited apparent inflammation indicated by immune cell infiltration, possibly due to bacterial infection and rapid dehydration. On the contrary, the inflammation was significantly relieved in the BDM group (Fig. 6F). Subsequently on day 7, we observed the initial epithelial tissue formation, and the BDM group demonstrated more well-defined granulation tissue regeneration with the smallest gap between the demises. Last, after 14 days, the wound treated with our BDM exhibited remarkable skin regeneration with consecutive skin surface and unnoticeable scar formation (Fig. 6F). The quantitative analysis of epidermal thickness showed that the BDM group exhibited the greatest epidermis thickness (~23 μm) (Fig. 6G), which closely resembled the healthy epidermis (*46*). In addition, the BDM group demonstrated enhanced hair follicle density (~8.3/mm^2^), which was 5.66- and 2.44-fold higher compared to the blank and fibrin glue groups, respectively (Fig. 6H). This boosted hair follicle density comparable to that of the normal skin (~9.7/mm^2^) (*47*) indicates that our BDMs could induce reconfiguration of the remodeling process while suppressing scar formation.

Moreover, we performed CD31 immunofluorescence staining to evaluate the newly formed vessels (*48*). The wounds treated with the BDM presented significantly higher CD31 expression with largest blood vessel formation, indicating the significantly nourished angiogenesis during wound healing (fig. S21, A and B). We next adopted Masson’s trichrome staining to examine the collagen organization (collagen is a major skin component) on day 14 (fig. S22, A and B). We found that the BDM group exhibited a significant increase in collagen deposition with well-organized fibroplasia, suggesting that BDMs can effectively promote ECM deposition. To further validate the suppressed scar formation, we conducted immunofluorescence staining for type I and type III collagens (Col-I and Col-III) (fig. S22, C and D). The ECM of scar tissue is primarily composed of Col-I, whereas the fetal scarless healing process is characterized by a higher abundance of Col-III with low Col-I/Col-III ratio (*49*). Accordingly, interventions that target excessive Col-I deposition and promote Col-III synthesis hold significant potential for reducing scarring. Notably, our BDM exhibited a significantly reduced Col-I expression and a corresponding increase in Col-III expression compared to the other groups (fig. S22E). It also demonstrated the significantly lower Col-I/Col-III ratio at approximately 1.3 (comparable to the normal skin, ~1.2) (*50*), indicating the substantially inhibited scar formation (fig. S22F). Together, these findings collectively demonstrated that our BDMs have tremendous potential to empower scarless wound healing, making them highly relevant for clinical translation and application.

### Mechanism elucidation of the hierarchically programmed scarless wound healing

**Fig. 7.**
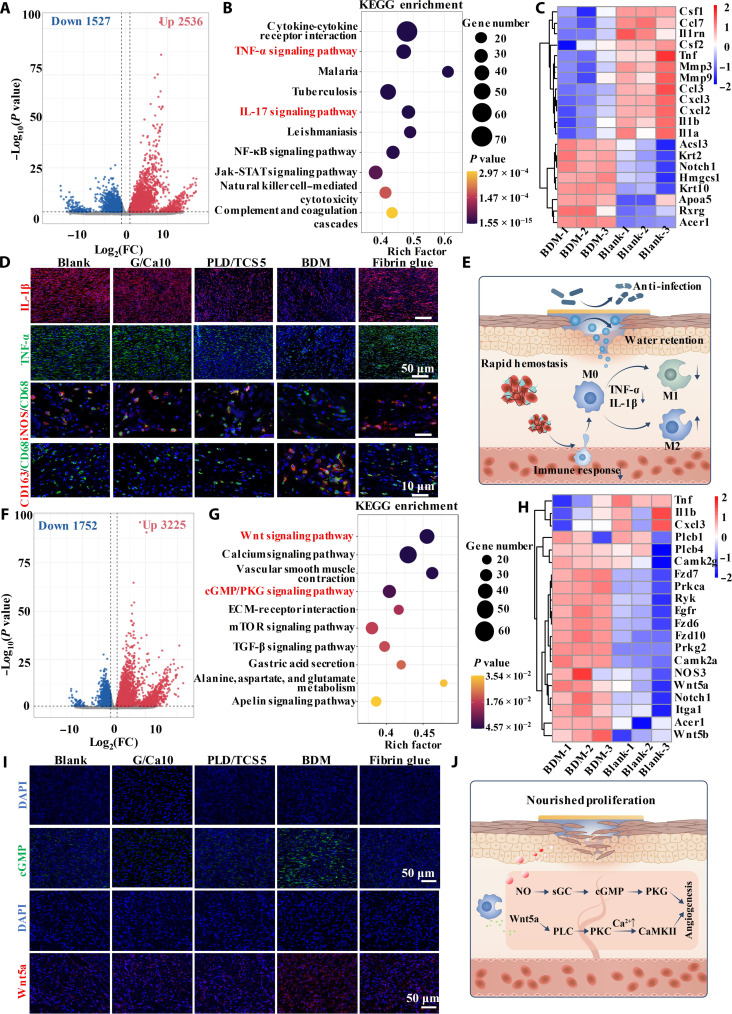
Transcriptomic analysis of the BDMs on hierarchically programmed scarless wound healing. (**A**) Volcano plots showing DGEs between the BDM group and the blank group on day 3. FC, fold change; NF-κB, nuclear factor κB; Jak, Janus kinase; STATsignal transducers and activators of transcription. (**B**) Down-regulated enriched KEGG pathways and (**C**) gene expression heatmap related to the activated pathways. (**D**) Immunofluorescence staining of IL-1β, TNF-α, iNOS (M1 marker), CD163 (M2 marker), and CD68 (M0 marker) on day 3. (**E**) Schematic demonstrating the BDMs creating rapid hemostasis and moist and sterile environment to synergically suppress the early inflammation with increased M2 macrophage polarization. (**F**) Volcano plots showing DGEs between the BDM group and the blank group on day 7. (**G**) Up-regulated enriched KEGG pathways and (**H**) gene expression heatmap related to the activated pathways. mTOR, mammalian target of rapamycin. (**I**) Immunofluorescence staining cGMP and Wnt5a on day 7. DAPI, 4′,6-diamidino-2-phenylindole. (**J**) Schematic demonstrating the BDMs to enhance angiogenesis with coactivation and coordination of the cGMP/PKG-Wnt/Ca^2+^ signaling pathways.

To further elucidate the potential mechanism of the enhanced scarless wound healing empowered by our BDMs with hierarchically programmed healing cascade, we collected wound tissues and performed transcriptomic analysis (BDM group versus blank group) on day 3 and day 7, targeting the inflammation and proliferation stages respectively (*51*). On day 3, a wide differential gene expression (DGE) was observed, and the volcano plots additionally demonstrated 2536 up-regulated and 1527 down-regulated genes (Fig. 7A). We then collected the DGEs to perform gene ontology (GO) database analysis, including biological process, cellular component, and molecular function. We found that various GOs related to immune response and inflammation (e.g., inflammatory response, response to bacterium, and immune response) were significantly down-regulated, indicating that our BDMs could alleviate the wound inflammation after implementation, which is critical for scarless wound healing (fig. S23A). Meanwhile, the GO terms also presented the up-regulation of the epidermis development and keratinocyte differentiation, indicating the transition of inflammation to proliferation (fig. S23B) (*52*). We then performed Kyoto Encyclopedia of Genes and Genomes (KEGG) analysis, exhibiting significant down-regulation of the TNF-α and interleukin-17 (IL-17) signaling pathways, which were associated with proinflammatory M1-type macrophage polarization (Fig. 7B) (*26*). Moreover, we observed the up-regulation of the peroxisome proliferator-activated receptor signaling pathway, which was related to prohealing M2 macrophage activation (fig. S24) (*49*). Furthermore, the heatmap depicting the differentially expressed genes in these pathways exhibited significant down-regulation (e.g., MMP3, TNF, IL-1α, IL-1β, and CCL3) or up-regulation (e.g., Krt2, Krt3, Rxrg, and Acer1) (Fig. 7C). During hemostasis, the activated platelets could secrete immune mediators (e.g., IL-8 and transforming growth factor–β) to recruit immune cells (e.g., macrophages) and, thus, to evoke the immune response (*53*). However, the overactivation of platelets in uncontrollable bleeding could cause the excessive secretion of inflammatory cytokines to provoke exaggerated immune response accompanied with chronic inflammation (*54*). It has been demonstrated that rapid hemostasis with lower immune mediators released by platelets could reduce the inflammatory response for scarless wound healing (*55*, *56*). Moreover, the wound dehydration and infection could also stimulate the proinflammatory cytokines production (e.g., IL-1 and TNF-α) with stalled inflammation phase leading to scar formation (*57*, *58*). Thus, our BDM with maintenance of moist and sterile environment could potentially tune the level of proinflammatory cytokines and advance the inflammation to proliferation. To this end, we validated the key inflammatory factor expression (e.g., IL-1β and TNF-α) and the macrophage polarization through immunofluorescence staining on day 3 (Fig. 7D). We found that the BDM groups presented significantly inhibited IL-1β and TNF-α expression, confirming the suppressed inflammatory microenvironment (Fig. 7D and fig. S25). In addition, the immunofluorescence staining of the inducible nitric oxide synthase (iNOS) (M1 marker), CD163 (M2 marker), and CD68 (M0 marker) revealed that our BDMs could effectively promoted the macrophage M2 polarization with significantly decreased M1 ratio (10.6 ± 2.3%), increased M2 ratio (17.6 ± 1.32%), and the lowest M1/M2 ratio (0.59 ± 0.11) (Fig. 7D and fig. S26). In all, our BDMs creating the rapid hemostasis and moist, breathable sterile environment could suppress the early inflammation with increased M2 macrophage polarization and program the healing cascade from the inflammation to proliferation (Fig. 7E).

On day 7, the volcano plots presented 3225 up-regulated and 1752 down-regulated genes (Fig. 7F). In GO analysis, we observed several up-regulated terms related to skin tissue regeneration such as epithelial cell morphogenesis, hair follicle development, and epidermis development (fig. S27). The KEGG analysis further revealed the up-regulation of Wnt signaling pathway and cGMP/PKG signaling pathway, which have demonstrated critical roles in angiogenesis promotion to boost the proliferative wound healing (Fig. 7G). The heatmap showing the up-regulation of key differentially expressed genes (e.g., Wnt5a, Fzd7, Prkca, Prkg2, and NOS3) further confirmed the activation of the pathways (Fig. 7H). Previously, we showed that the cGMP/PKG pathway is decisive for the boosted angiogenesis and could be mediated by NO production (*17*). Thus, the NO generated by our BDMs could sequentially activate the soluble guanylate cyclase (sGC), cGMP, and PKG for enhanced angiogenesis. In addition, we found the significantly up-regulation of the Wnt5a, phospholipase C (PLC), protein kinase C (PKC), and Ca^2+^/calmodulin-dependent protein kinase II (CaMKII) (Fig. 7H). The Wnt5a is the typical ligand of the noncanonical Wnt/Ca^2+^ pathway with critical role in regulating the endothelial cell fate (*59*). Previously, it has been reported that Wnt5a could be released by the polarized M2 macrophages to activate the downstream PLC and PKC to increase intracellular Ca^2+^ concentration and further activate the CaMKII for enhanced angiogenesis (*60*, *61*). The cGMP/PKG pathway may cross-talk with the Wnt/Ca^2+^ pathway due to the increased intracellular Ca^2+^ modulated by the Wnt/Ca^2+^ pathway, which may further regulate the eNOS activity to increase the NO production. Thus, we validated the expression of cGMP and Wnt5a in cGMP/PKG and Wnt/Ca^2+^ pathway, respectively (Fig. 7I). We observed an up-regulation of cGMP expression in the BDM groups (4.38- and 3.92-fold compared to the blank and fibrin glue groups), indicating that the BDMs can activate the cGMP/PKG signaling pathway in vivo for boosted angiogenesis (fig. S28). In addition, we found a significantly higher expression of Wnt5a in the BDM group (5.65- and 2.09-fold compared to the blank and fibrin glue groups), which could be attributed to the facilitated M2 macrophage polarization (fig. S28). The higher Wnt5a expression was expected to subsequently activate the Wnt/Ca^2+^ pathway to promote the wound healing. We thus theorized that the enhanced angiogenesis during the proliferation stage could be resulted from the coactivation and coordination of the cGMP/PKG-Wnt/Ca^2+^ signaling pathways (Fig. 7J).

In all, we demonstrated that our BDMs could promote scarless wound healing by programming the stepwise healing cascade by creating a hemostatic, moist, sterile, and angiogenic microenvironment. It could not only suppress the inflammatory TNF-α pathways with increased M2 macrophage polarization to facilitate the transition of inflammation to proliferation but also stimulate the angiogenesis during the proliferation by coactivation and coordination of the cGMP/PKG-Wnt/Ca^2+^signaling pathways.

### BDMs enhanced wound healing in full-thickness porcine skin wound model

**Fig. 8.**
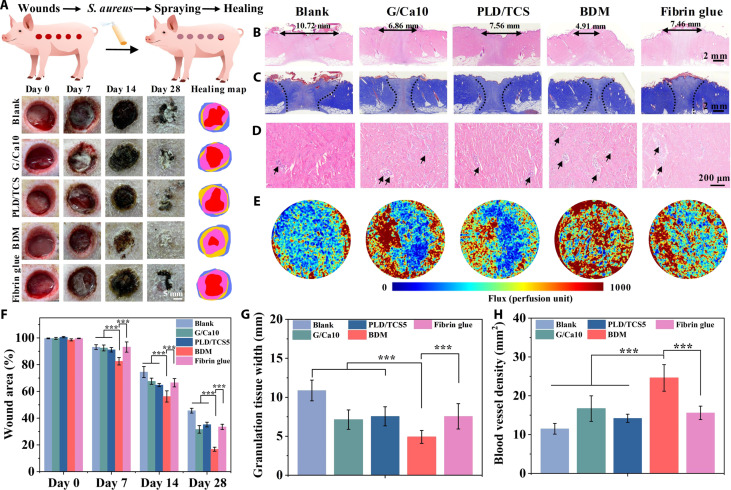
BDMs enhanced wound healing in full-thickness porcine skin wound model. (**A**) Schematic illustration showing the construction of *S. aureus*–infected full-thickness porcine skin wound model and the representative wound images treated with different wound masks. (**B**) H&E staining of wound sections after 28-day treatment. The black arrow lines indicate the width of granulation tissue. (**C**) Masson’s trichrome staining of wound sections after 28-day treatment. The dashed lines indicate the wound areas. (**D**) Luminal structure formation and (**E**) laser Doppler perfusion images of the healing area. The black arrows indicate the vessels. Quantification of (**F**) wound area percentage, (**G**) granulation tissue width, and (**H**) blood vessel density of the healing area. Sample size *n* = 5 for all experiments by a one-way or two-way ANOVA with a Tukey’s post hoc test for multiple comparisons. Data are presented as means ± SD. ***P < 0.001 is considered statistically significant.

To provide more insight into the therapeutic wound healing efficacy of our BDMs with closer human skin resemblance, we further performed the full-thickness porcine wound model, which is widely recognized for its physiological and anatomical similarities to the human skin (*46*). We found that our BDM substantially accelerated wound closure throughout the healing process. By day 7, the release of TCS from the BDMs significantly reduced the infection on the wound sites, decreasing the inflammation reaction (Fig. 8A). In addition, the wound area of the BDM group decreased to around 83.6%, compared to 95.1% in the blank group and 91.2% in the fibrin glue group. By day 28, wound closure in the BDM group was nearly complete, whereas 50% of the wound in the control group remained unhealed. Moreover, histological H&E staining analysis showed that the width of the granulation tissue in the BDM group was significantly narrower than that in other groups, indicating minimal scar hyperplasia in the newly formed tissue (Fig. 8, B and G). Furthermore, Masson’s trichrome staining revealed the most extensive collagen deposition in the BDM group compared to other groups, demonstrating the effectiveness of BDMs to facilitate the reconstruction of collagen during wound healing (Fig. 8C and fig. S29A). To further analyze the formation and functionality of the newly formed blood vessels, we quantified RBC perfusion with luminal structure formation using H&E staining (Fig. 8D). We found that the BDM group had a higher number of newly formed vessel lumen structures, 2.13- and 1.57-fold compared to control and fibrin glue groups, respectively, highlighting its superior angiogenic capability (Fig. 8H). Moreover, to quantitatively assess blood flow within the healing tissue, we conducted laser Doppler perfusion imaging (Fig. 8E). We found that the BDM group exhibited the highest blood flow flux with 1.74-fold higher to fibrin glue, indicating the reconstruction of functional blood flow (fig. S29B). These results collectively demonstrated that our BDMs not only promote the formation of endothelial cell structures but also enhance the development of fully functional, perfused vascular networks, which are crucial for successful wound healing.

## DISCUSSION

In this project, we develop a sprayable BDM with rapid autophasing and hierarchical programming for scarless healing of extensive wounds. The sprayable BDMs constitute of photocrosslinkable hydrophobic PLD polymer and hydrophilic GelMA hydrogel. The two phases swiftly autophase into bilayered structure through spontaneous water/oil separation. After photocrosslinking, the BDMs demonstrate strong interfacial bonding, robust tissue adhesion, and excellent adaptiveness to joint movement. Moreover, our BDMs programmatically stimulate the dynamic healing cascade. The bottom GelMA layer rapidly releases Ca^2+^ for hemostasis, while the top PLD layer maintains a moist, breathable, and sterile environment, thus synergistically suppressing the inflammatory TNF-α pathway for increased M2 macrophage polarization. Our BDMs also coactivate and coordinate the cGMP/PKG-Wnt/Ca^2+^ signaling pathways to enhance the vascular reconstruction and empower scarless wound healing.

To the best of our knowledge, our sprayable BDMs represent the first attempt to engineering an in situ forming, sprayable bilayer wound mask that achieves spontaneous autophasing via the intelligent control of water/oil phase separation. This biomimetic bilayer mask, with its rapid application ability and simple implementation process, represents an unprecedented advancement in the field of wound care technology. It also provides a facile scientific concept for wound mask design to dynamically program the healing cascade for scarless wound healing. This approach demonstrates a facile integration of rapid hemostasis, maintenance of a moist, breathable and sterile environment, and angiogenesis nourishment to inhibit inflammation and activate prohealing pathways. From a clinical perspective, our BDMs only contain inherent or metabolizable elements of the human body and can be readily used through simple mixing without complex chemical intervention. The remarkably simplified yet effective implementation procedure is of great value in time-sensitive emergency medical interventions. Notably, compared with existing hydrophobic/hydrophilic bilayered wound matrices such as Integra Bilayer Matrix Wound Dressing, which comprises a bottom hydrophilic layer of collagen and glycosaminoglycan and an upper hydrophobic layer of polysiloxane, our BDMs demonstrate distinctive features and benefits. These include a compatible material composition (the bottom and top layer materials can cophotocrosslink with each other due to the presence of C═C in both materials) that strengthens the interfacial bonding of the two layers, fitness to irregular shape of wounds due to the sprayability, superior tissue adhesion, capability for a personalized design, and enhanced therapeutic efficacy with rapid hemostasis and nourished angiogenesis. Together, our BDMs have significant translational implications and the potential to greatly benefit patients with extensive wounds.

Despite the superior potential of BDMs, some limitations of our research should be acknowledged. The autophasing of the hydrophobic PLD polymer and hydrophilic GelMA hydrogel relies on the distinct densities of the two components. Consequently, the application of our BDMs needs to be applied horizontally. Future studies will be focusing on how to solidify the wound spray as soon as it reaches the skin to broaden its applications under various circumstances.

## MATERIALS AND METHODS

### Materials synthesis and characterization

GelMA with different C═C modification degrees was synthesized using our previous protocol (*17*, *62*). Briefly, 15% (w/v) of gelatin (Sigma-Aldrich, Hong Kong) was firstly dissolved in phosphate-buffered saline (PBS; Thermo Fisher Scientific, Hong Kong) at 60°C, followed by the addition of various methacrylic anhydrides (Sigma-Aldrich, Hong Kong) for G30, G60, and G90 respectively. The resultant product was dialyzed for impurity removal. The synthesized GelMA was evaluated by the FTIR (Bruker, USA) and the ^1^H NMR (Jeol, Japan). The PLDs with different molecular weights (P*m*L*n*D) were also synthesized following our own protocol (*18*). For P7L2D, 40 g of propylene glycol (Macklin, China) was firstly reacted with the 23 g of lactide (Macklin, China) for the synthesis of intermediate product of poly(lactide-*co*–propylene glycol–*co*-lactide). Then, 4.22 g of methacryloyl chloride (Sigma-Aldrich, Hong Kong) and 4.05 g of triethylamine (Sigma-Aldrich, Hong Kong) were diluted in dichloromethane (Macklin, China) and added in the reaction system. The resultant product was characterized by FTIR and ^1^H NMR analysis.

The working solution of GelMA was prepared by dissolving pure GelMA in DI water (10%, w/v) with addition of lithium phenyl-2,4,6-trimethylbenzoylphosphinate (0.3 wt %; SunP, Beijing) as photoinitiator due to its hydrophilicity (*17*). The working solution of P*m*L*n*D was prepared by supplementation of 4.5 wt % of hydroxyethyl methacrylate (Sigma-Aldrich, Hong Kong) and 0.5 wt % of phenylbis(2,4,6-trimethylbenzoyl)phosphine oxide (Irgacure 819, BASF, Germany) as photoinitiator due to its hydrophobicity (*18*). We evaluated the viscosity and cross-linking kinetics of the GelMA and P*m*L*n*D by the rheometer (Anton Paar, Austria). Briefly, the viscosity was tested under rotational mode with 1 to 100 s^−1^ of shear rates. Next, the rheological monitoring of the storage modulus was tested with 1% shear strain and 10-Hz frequency to quantify the cross-linking kinetics. The degradation of the GelMA and P*m*L*n*D was performed with reported literature in CL solution (0.5 U/ml) and DI water, respectively (*62*). The mass changes were recorded by the high-precision balance at predetermined time points. Then, we adopted the universal testing machine to test the mechanical properties of GelMA and PLD based on ASTM D882 standard (*63*). Each sample (20 mm by 5 mm) was stretched until failure with a rate of 1 mm/min at room temperature.

### Preparation and characterization of the sprayable BDMs

For the preparation of calcium chloride (CaCl_2_; Sigma-Aldrich, Hong Kong) incorporated working solutions, 5, 10, and 15 wt % of CaCl_2_ was added in the prepared GelMA solution followed with thoroughly mixing (denoted as G/Ca5, G/Ca10, and G/Ca15). To bestow antibacterial properties upon the sprayable BDMs, we incorporated TCS into the PLD. Briefly, after the preparation of the PLD working solution, different concentrations of TCS (e.g., 2.5, 5, and 10 wt %) were incorporated, followed with thoroughly mixing (denoted as PLD/TCS2.5, PLD/TCS5, and PLD/TCS10). We then mixed the working solution of GelMA and PLD with a 1:1 volume ratio to prepare the sprayable BDMs to balance the structural integrity and biological functionality of the two layers, ensuring optimal performance in promoting wound healing. After implementation, the sprayed materials were cross-linked under blue light irradiation (405 nm, 5 mW/cm^2^) for 100 s (*64*). The microstructure of the cross-linked materials was characterized by the SEM (Zeiss, Germany). Then, the strength of interface was characterized by shear test. After the injection and cross-linking of 1 ml of the mixed materials in 2-cm by 2-cm mold, the formed bilayered scaffold was tightly glued to the glass slides with continuous compression to ensure good contact. Then, the shear bonding strength was measured using a universal testing machine (MTS, USA) by stretching the glass slides at a constant rate of 1 mm/min until materials failure (*65*). The tissue adhesion capacity was evaluated by standardized burst pressure testing with our self-developed apparatus (*66*). Briefly, a cleaned porcine skin membrane (Ø = 3 cm) with an incision (Ø = 1 mm) in the center was fixed to the measurement tool connecting a syringe pump filled PBS. Then, 100 μl of different solutions were sprayed onto the incision, followed with in situ photocrosslinking. Pressure was applied on the closed incision site through pumping PBS at a flow rate of 2 ml/min. The peak pressure before material failure was recorded by high-speed pressure transducer to indicate the burst release pressure. The lap shear test was performed with the previous protocol (*67*). The samples were inserted uniformly between two fresh porcine tissues with gluing. Then, the universal mechanical tester was applied to pull the porcine tissues (1 mm/min) for the evaluation of adhesion strength. Shear strength = *F*/*A*, where *F* is the pulling force and *A* is the adhesive area. For scratch test, we created a straight-line scratch on the surface of the materials with a sterile scalpel with consistent scratch depth and observed the scratch area by optical microscope (Olympus, Japan) (*68*).

### In vitro hemostatic evaluation of the sprayable autophasable BDMs

In vitro clotting time assay procedures were performed in accordance with a previous protocol with commercial fibrin glue (Tisseal, Baxter, USA) as control (*69*). Briefly, the BDMs were prepared by different Ca^2+^-loaded GelMA solutions (100 μl) with naked PLD solution (100 μl), followed with in situ cross-linking in 24-well plate. Then, 100 μl of fresh rabbit blood (Bersee, Beijing) was added to the 24-well plate. At different predetermined time points, each well was gently washed with saline solution. The clotting time was determined by the time point when the blood in the well formed a uniform clot. For the preparation of thrombin-free fibrin glue, 100 mg of fibrin was dissolved in 1 ml of CaCl_2_ solution (10 wt %), followed with gentle agitation and incubation at 37°C for 12 hours to form a gel (*25*). Then, we evaluated the Ca^2+^ release kinetics. Briefly, the prepared BDMs were immersed in 20 ml of DI water at 37°C. At different predetermined time points, 50 μl of solution was collected for Ca^2+^ concentration evaluation using inductively coupled plasma optical emission spectrometry (Thermo Fisher Scientific) (*17*). Then, RBC (SolarBio, Beijing) absorption was assessed (*24*). RBC solution (200 ml) was dripped on 10 mg of samples at 37°C for 1 hour. After rinsing five times with PBS, the samples were put in 5 ml of DI water for 1 hour to lyse RBC. Then, 100 μl of lysed liquid was measured by microplate reader (BioTek, USA) at 541 nm. The percentage of adsorption was quantified by the following equation: percentage of adhered RBC (%) = (optical density sample)/(optical density reference value) × 100% (*70*). The platelet adhesion was measured by lactate dehydrogenase kit (Beyotime, Beijing) (*24*). Briefly, platelet-rich plasma was generated by centrifuging the whole blood at 200*g* for 15 min, and then 100 μl of platelet-rich plasma was dripped on different BDMs at 37°C. The platelets were lysed by 1% Triton X-100, and the lactate dehydrogenase was evaluated according to the manufacturer’s instructions.

### In vivo hemostatic evaluation of the sprayable autophasable BDMs

Rat tail amputation model and hemorrhaging liver model (male SD rats, ~200 g) were used to investigate the in vivo hemostatic capacity of the sprayable mask with the permission from the Ethics Committee of the Hong Kong Polytechnic University Commercial (22-23/362-BME-R-GRF). For the hemorrhaging liver model, we exposed and dissected the liver with a needle after the rat anesthetization with 1% pentobarbital (Sigma-Aldrich, Hong Kong) (*24*). Then, 200 μl of different BDMs were implemented and photocrosslinked. Afterward, a preweighed filter paper was placed beneath the bleeding site for the observation of the hemostatic time and quantification of the relative blood loss until the equilibrium state was reached. To further validate the hemostatic capacity of the BDMs, we performed the rat tail amputation model. After the rats were anesthetized, 30% rat tails were cut (*71*). Immediately, 100 μl of different BDMs were deployed over the bleeding site and photocrosslinked. We also recorded the hemostatic time and quantified the relative blood loss.

### Evaluation of moist, breathable, and sterile environment using BDMs

The WVTR of the samples was measured following the previous protocol (*72*). Briefly, round samples with 30 mm in diameter were used to seal the mouth of bottle with 29 mm in diameter containing 20 ml of DI water. The bottles were placed in an incubator at 37°C and with 35% humidity. At different time points, water loss was weighted for the WVTR calculation. Oxygen permeability was performed with the reported Winkler method (*32*). All samples (1 cm by 1 cm) were secured on the opening of round bottles half-filled with DI water using a Teflon tape. After 24 hours, the dissolved oxygen levels were measured using an oximeter. The open bottle and sealed bottle were used as the positive control and negative control, respectively. Water contact angle measurement was performed by a contact angle analysis system (Krüss, Germany) using the sessile drop method. Each sample surface was delivered with 10-μl drop of DI water at room temperature for the water contact angle evaluation. The antifouling property of different materials was evaluated with the FITC-BSA as a model protein (*73*). Briefly, the round samples (Ø = 8 mm) were incubated with a FITC-BSA solution (0.5 mg/ml). After thoroughly rinsing with PBS, the fluorescence signals were detected by a fluorescence microscope (Nikon, Japan).

To bestow antibacterial properties of the sprayable BDMs, we further incorporated TCS into the PLD layer. Briefly, after the preparation of the PLD working solution, different concentrations of TCS (e.g., 2.5, 5, and 10 wt %) were incorporated, followed with thoroughly mixing. We firstly evaluated the biocompatibility of the prepared samples (Ø = 10 mm) in which the NIH/3T3 cells (American Type Culture Collection, USA) were used following the ISO 10993 standard (*74*). Briefly, all samples (Ø = 10 mm) were soaked in the full culture medium for 24 hours at 37°C. Then, the extracted culture medium was used to seed the cells in the 24-well plate at a density of 1 × 10^4^ cells/cm^2^. After 1, 2, and 3 days of incubation, the Live/Dead assay kit (Thermo Fisher Scientific, Hong Kong) and the CCK-8 (Cell Counting Kit-8) kit (Sigma-Aldrich, Hong Kong) were used to evaluate the cell viability and cell proliferation, respectively (*75*). The antibacterial activity of the samples was examined via inhibition zone assay and spread plate method with Gram-negative *E. coli* and Gram-positive *S. aureus* (*51*)*.* For inhibition zone assay, after the preparation and sterilization of Luria-Bertani (LB; Sigma-Aldrich, Hong Kong) medium, 1 ml of bacterial solution [1 × 10^5^ colony-forming units (CFU)/ml] was inoculated on the LB plate, followed with placing different samples in the center of the plate. After culturing at 37°C for 24 hours, the inhibition zone was recorded to evaluate the antibacterial effect. The antibacterial activity of the prepared samples was also tested by spread plate method. Briefly, 1 ml of bacterial solution (1 × 10^5^ CFU/ml) was incubated with the degradation medium after 14 days at 37°C for 24 hours. Then, the bacterial solution was diluted (1:10,000) and plated onto LB plates for 24 hours incubation at 37°C. The formed CFU on the LB plates were also counted. The TCS release rate of the samples (Ø = 10 mm) was tested by immersing in 10 ml of DI water at 37°C. At the same time, the TCS concentration was characterized using ultraviolet-visible analysis at 281 nm.

### Evaluation of nourished angiogenesis of the sprayable autophasable BDMs

To validate the wound nourishment capacity of our BDMs, we prepared different samples (Ø = 5 mm by 3 mm) with the CaCl_2_ incorporation in GelMA layer (e.g., G/Ca5 and G/Ca10) and naked PLD layer. After immersing in 1 ml of PBS for 10 min (simulating the rapid hemostasis with massive Ca^2+^ release), we collected the samples and further test the release kinetics of the residual Ca^2+^ and l-arginine in the presence of CL (0.5 U/ml and denoted as CL+) (*62*). The samples without the CL were used as control (denoted as CL−). We tested the Ca^2+^ release by inductively coupled plasma optical emission spectrometry (Agilent, USA) and the l-arginine by l-arginine assay kit (Abcam, Hong Kong) following the manufacturer’s protocol (*48*). To evaluate the nourishing and angiogenesis effect of the degradation product, we prepared the conditioned medium based on the ISO 10993 standard. Briefly, different samples (Ø = 5 mm by 3 mm) were immersed in 1 ml of RPMI 1640 medium (Gibco, Hong Kong) with the addition of CL without fetal bovine serum. After incubation for 24 hours at 37°C, the medium was collected and heated to 60°C for 10 min to deactivate the CL. Last, the cooled medium was supplemented with 10% fetal bovine serum to prepare the full conditioned medium. We then used the full conditioned medium for the in vitro angiogenesis evaluation with the use of HUVECs (*48*).

We firstly evaluated the effect of the conditioned medium on the angiogenesis of HUVECs (1 × 10^4^ cells/cm^2^) (*48*). The relative expression of eNOS was measured after 3 days of incubation by real-time polymerase chain reaction (*17*). The total NO generation was quantified using total nitric oxide assay kit (Beyotime, Beijing). For short-term NO release (within 2 hours), we used DAF-FM DA (NO probe) for NO production detection in cells (*76*). The HUVEC migration behavior was tested by in vitro scratch wound assay. HUVECs (5000 cells/cm^2^) were seeded in six-well plates. Then, a scratch was made on the cell monolayer of each disc using a 200-μl size plastic pipette tip. Then, each disc was washed with PBS and changed with different conditioned media. After 24 hours, cells were stained with crystalline violet, and cell migration was observed. To further evaluate the angiogenesis efficacy of BDMs, we performed the tube formation assay (*17*). Briefly, 100 μl of chilled (4°C) Matrigel (Corning, USA) was used to coat the 24-well plate, followed with solidification under 37°C. Then, HUVECs (4 × 10^4^ cells/cm^2^) were seeded onto the Matrigel-coated 24-well plate and cultured with various conditioned media. After 2 and 6 hours, the HUVECs were stained by calcein acetoxymethyl for 10 min, and capillary-like structures were observed with a fluorescence microscope (Zeiss, Germany). The branch points of the capillary-like structures were quantified by ImageJ software with six randomly selected fields. For immunofluorescence staining of CD31, CD31 primary antibody and the corresponding secondary antibody (Abcam, Hong Kong) were used after the cell fixation. Cell nuclei were stained by 4′,6-diamidino-2-phenylindole (Sigma-Aldrich, Hong Kong). Images were captured by confocal microscopy (Leica, Germany) and quantified using ImageJ software.

### Biocompatibility evaluation of the BDM

NIH/3T3 cells (American Type Culture Collection, USA) were used to assess the in vitro biocompatibility of the BDM in accordance with the ISO 10993 standard (*74*). Briefly, different samples (Ø = 5 mm by 3 mm) were immersed in 1 ml of Dulbecco’s modified Eagle’s medium (Gibco, Hong Kong) for 24 hours to prepare conditioned medium. Subsequently, 2 × 10^4^ NIH/3T3 cells were seeded in 24-well plates and cultured with the conditioned medium. Bright-field images were captured at various time points to evaluate cell morphology. In addition, Live/Dead assay kit (Thermo Fisher Scientific, Hong Kong) and the CCK-8 kit (Sigma-Aldrich, Hong Kong) were used to quantify cell viability and proliferation, respectively.

### Full-thickness rat skin wound model

We established the rat *S. aureus*–infected full-thickness skin wound model for the in vivo therapeutic efficacy evaluation of the sprayable BDMs with permission from the Ethics Committee of the Hong Kong Polytechnic University (22-23/362-BME-R-GRF). Briefly, after the rat (male, ~200 g) anesthetization with 1% pentobarbital, the skin was exposed and disinfected (*77*, *78*). Then, four circular full-thickness wounds (Ø = 10 mm) were created on the dorsum with the inoculation of *S. aureus* suspension (0.2 ml, 10^8^ CFU/ml). Afterward, we deployed different materials onto the defects including the G/Ca10, PLD/TCS5, and BDMs. The commercial fibrin glue was used as control. At certain time points (e.g., 3, 7, and 14 days), equidistant photos of the healing wounds were taken for wound closure evaluation. After 3, 7, and 14 days, we also performed the histological analysis of the wounds. Four rats from each group were euthanized, and the wound samples were collected and fixed by formalin (TissuePro, Hong Kong). After being embedded in paraffin and sliced into sections, we performed the H&E staining to evaluate the wound healing process, while Masson’s trichrome was used to determine the deposition of collagen (*51*). Then, the CD31 and Col-I and Col-III immunofluorescence staining were additionally performed to evaluate the blood vessel regeneration and the scar formation (*26*).

### Transcriptomic evaluation of BDMs for scarless wound healing

We collected the wound tissue samples on day 3 and day 7 for RNA sequencing experiments. Briefly, total RNA was extracted from the skin tissue using TRIzol reagent (Thermo Fisher Scientific, Hong Kong), and RNA sequencing was conducted by NovaSeq 6000 (Illumina, USA). To identify the differentially expressed mRNAs, the “edgeR” R package was used for pairwise comparisons according to a fold change of >2 or <0.5. Furthermore, GO enrichment analysis and KEGG enrichment analysis were evaluated by the OmicStudio tools. Immunofluorescence staining for IL-1β, TNF-α, iNOS, CD68, CD163, Wnt5, and cGMP was performed following previously established methods (*17*, *26*, *79*). Raw data for transcriptomic analysis were deposited under National Center for Biotechnology Information (NCBI) BioProject PRJNA1033569.

### Full-thickness porcine skin wound model

The porcine model was established by the Huateng Biomedical Technology Limited (Guangzhou, China) with ethical approval (B202401-15). Panama minipigs with body weights ranging from 12 to 15 kg were used in this study (*n* = 5). All procedures were performed by qualified veterinary surgeons with strict adhesion to aseptic techniques. Before the surgical interventions, the hair on the minipigs’ backs was removed, and the skin was disinfected using ethanol. All surgical operations were conducted under general anesthesia. Five full-thickness wounds (Ø = 2 cm) were created on either side of the spine using a bistoury scalpel, maintaining a 4-cm gap between each wound. Subsequently, various materials were applied to the wounds, including G/Ca10, PLD/TCS5, and BDMs, with commercial fibrin glue serving as the control. Postoperatively, each minipig was housed individually and underwent daily monitoring. Wound images were captured, and the sizes of the wounds were measured at predetermined time points. On day 28, blood flow perfusion in the regenerated skin sites was assessed using a Doppler imaging system (MoorFLPI-2, Moor Instruments, UK). Following this perfusion assessment, the minipigs were euthanized, and the wound tissues were harvested for H&E and Masson’s trichrome histological analysis to evaluate the microscopic structure and collagen deposition within the regenerated tissue.

### Statistical analysis

All experiments were performed in triplicate unless otherwise indicated. All data were demonstrated as the means ± SD. Statistical differences were analyzed using one-way or two-way analysis of variance (ANOVA), followed by Tukey’s multiple comparisons test. *P < 0.05, **P < 0.01, and ***P < 0.001 were considered statistically significant.
