# Supplementary material for: Sprayable biomimetic double mask with rapid autophasing and hierarchical programming for scarless wound healing
Source: Sci Adv. 2024 Aug 14;10(33):eado9479. doi: 10.1126/sciadv.ado9479 (PMC11323895; doi:10.1126/sciadv.ado9479)
Supplement: Supplementary file 1 — Figs. S1 to S29 Legends for movies S1 and S2 [file sciadv.ado9479_sm.pdf]

Science Advances
AAAS

Supplementary Materials for

Sprayable biomimetic double mask with rapid autophasing and hierarchical programming for scarless wound healing

Yuhe Yang *et al.*

Corresponding author: Zijian Zheng, zijian.zheng@polyu.edu.hk; Bin Li, binli@suda.edu.cn;
Xin Zhao, xin.zhao@polyu.edu.hk

Sci. Adv. **10**, eado9479 (2024)
DOI: 10.1126/sciadv.ado9479

The PDF file includes:

Figs. S1 to S29
Legends for movies S1 and S2

Other Supplementary Material for this manuscript includes the following:

Movies S1 and S2

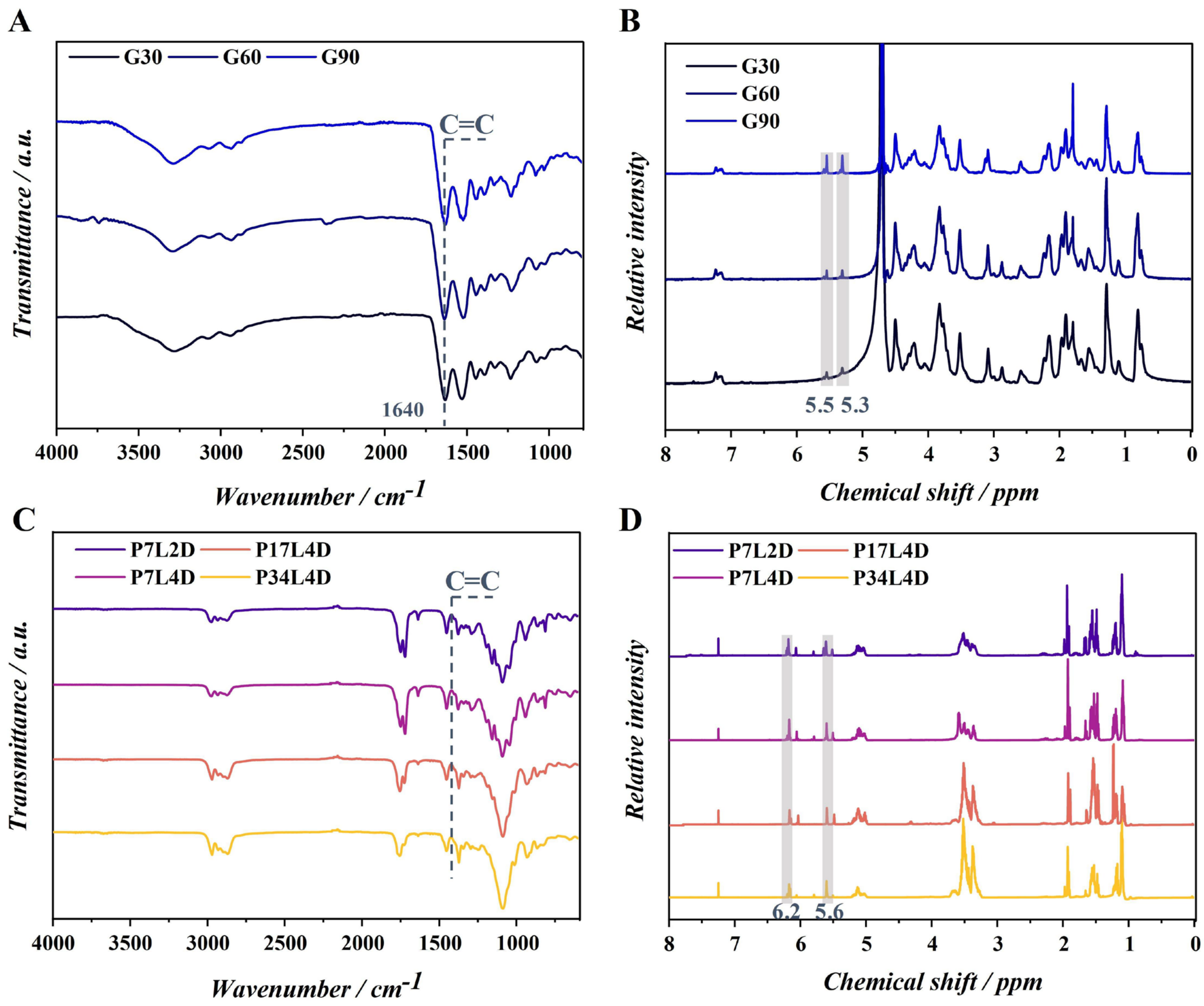

Fig. S1. (A) Fourier transform infrared (FTIR) and (B) ^{1}H nuclear magnetic resonance (^{1}H NMR) spectra of the synthesized gelatin methacrylate (GelMA) with 30%, 60% and 90% modification degree (i.e., G30, G60 and G90). (C) FTIR and (D)^{1}H NMR spectra of the synthesized PLD with different molecular weights (i.e., P7L2D, P7L4D, P17L4D and P34L4D).

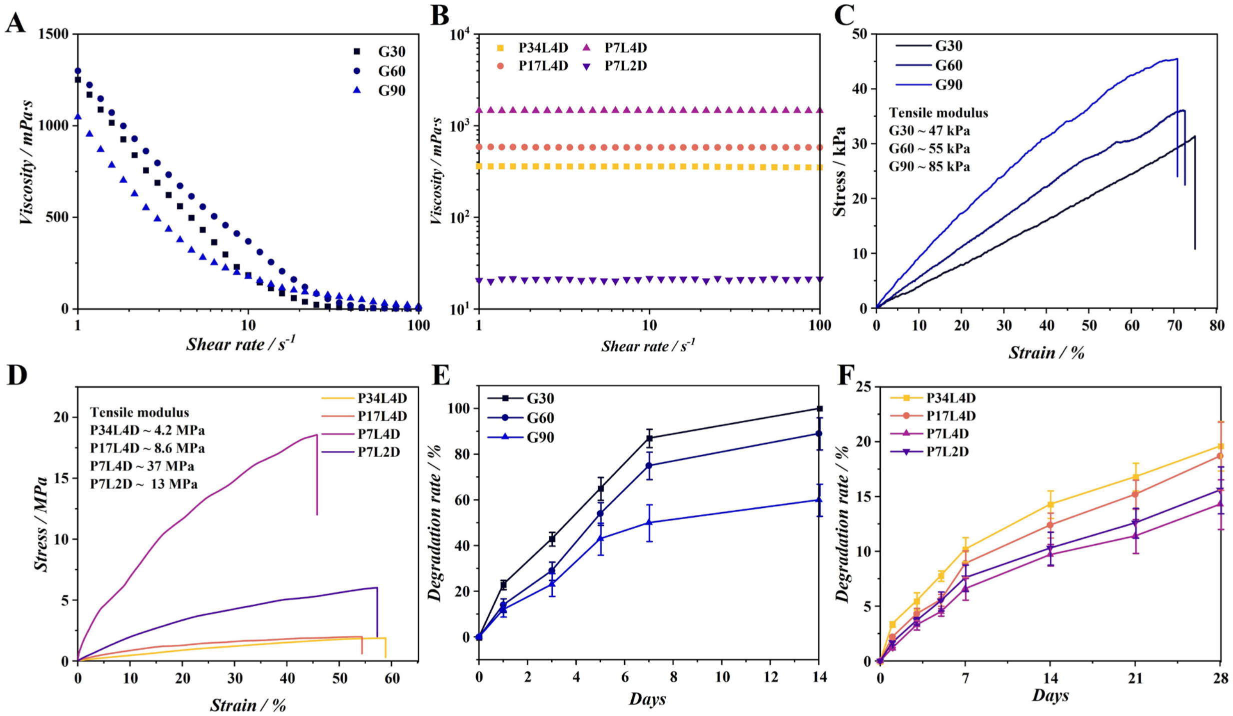

Fig. S2. Viscosity characterization of (A) different GelMA (i.e., G30, G60 and G90) and (B) PLD (i.e., P7L2D, P7L4D, P17L4D and P34L4D). The tensile stress-strain curve of the (C) different GelMA (i.e., G30, G60 and G90) and (D) PLD (i.e., P7L2D, P7L4D, P17L4D and P34L4D). The degradation evaluation of (E) different GelMA (i.e., G30, G60 and G90) and (F) PLD (i.e., P7L2D, P7L4D, P17L4D and P34L4D).

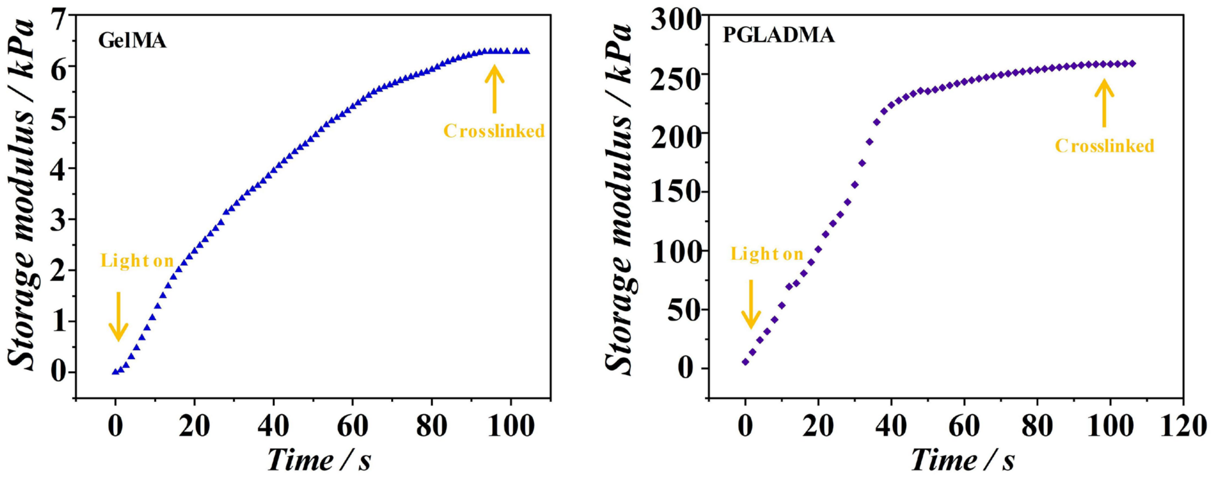

Fig. S3. Crosslinking kinetics evaluation of (A) G90 and (B) P7L2D via real-time storage modulus monitoring.

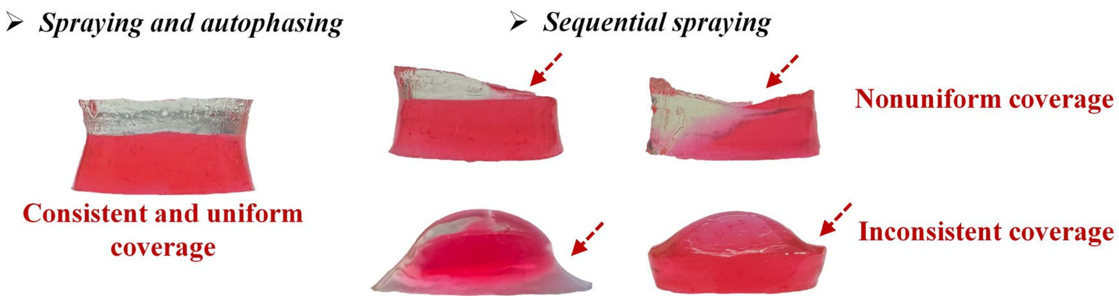

Fig S4. Various bilayer masks prepared by the spraying and autophasing and the sequential spraying processes.

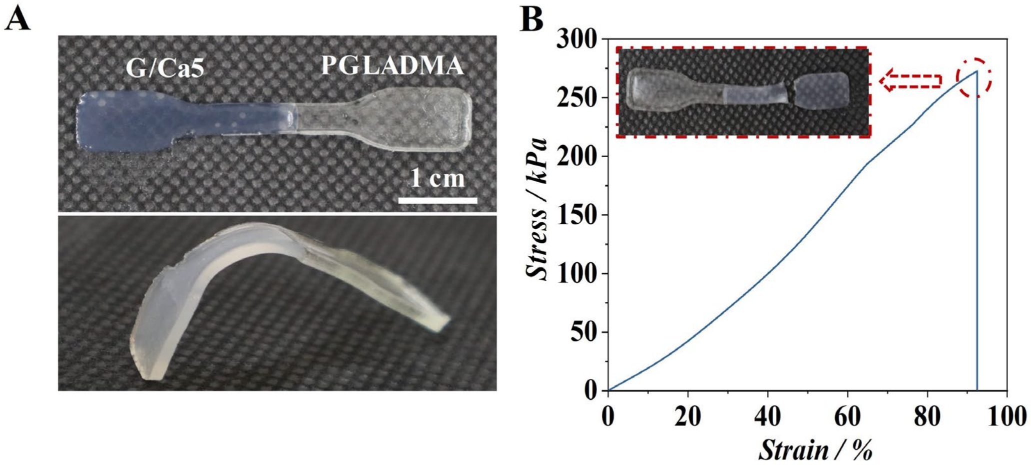

Fig. S5. (A) The standard tensile bar prepared by G/Ca5 and PLD. (B) The stress-strain curve of the prepared tensile bar. The inserted image indicated the crack position of the tensile bar.

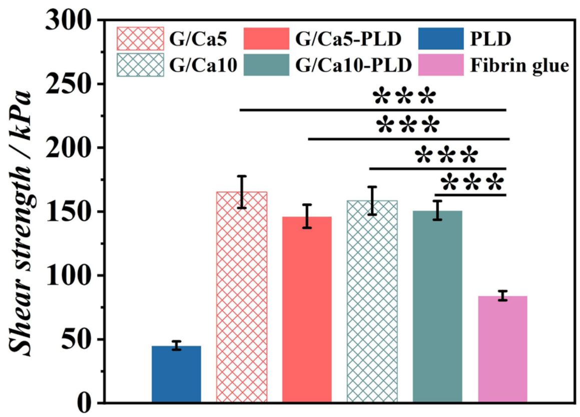

Fig. S6. The lap shear evaluation of our BDMs. Sample size $n = 3$ for all experiments by one-way ANOVA with a Tukey's post hoc test for multiple comparisons. Data are presented as mean ± SD. $^{**}p < 0.01$, $^{***}p < 0.001$ are considered statistically significant.

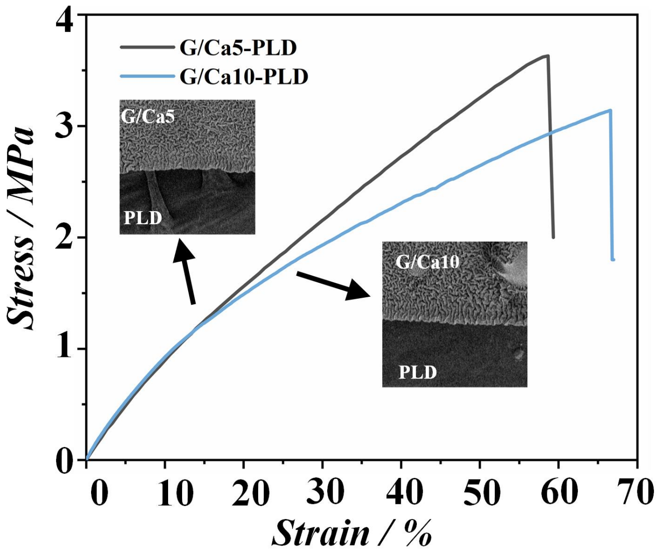

Fig. S7. The tensile stress-strain curve of the BDMs.

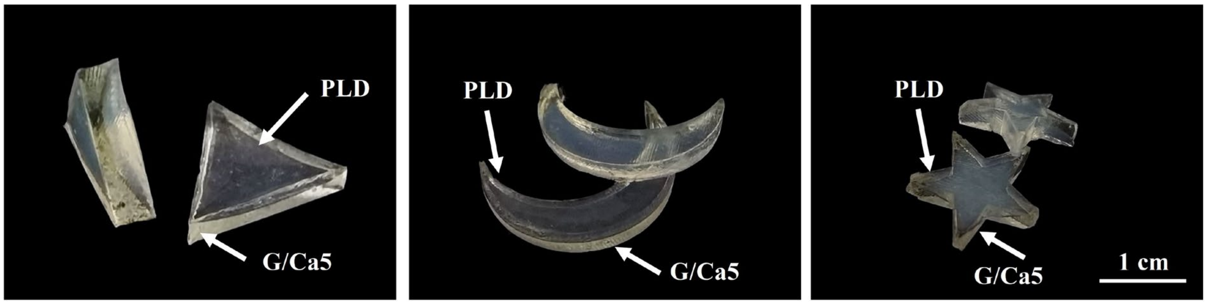

Fig. S8. The moldability of our BDMs to different shapes such as moon, star, and triangle mimicking various irregular wound geometries.

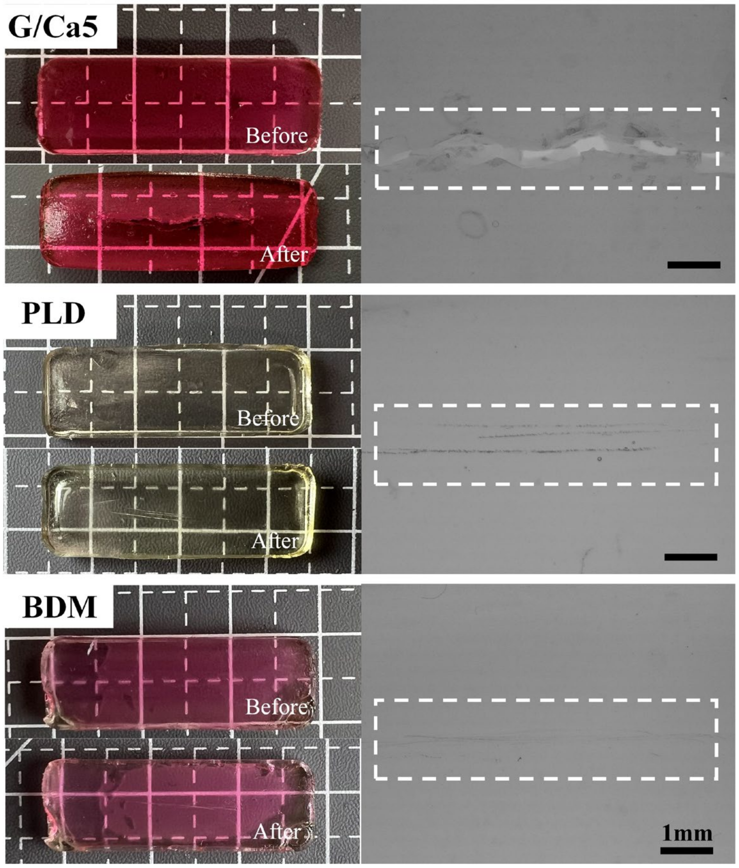

Fig. S9. Evaluation of stability of our BDMs against external mechanical scratches.

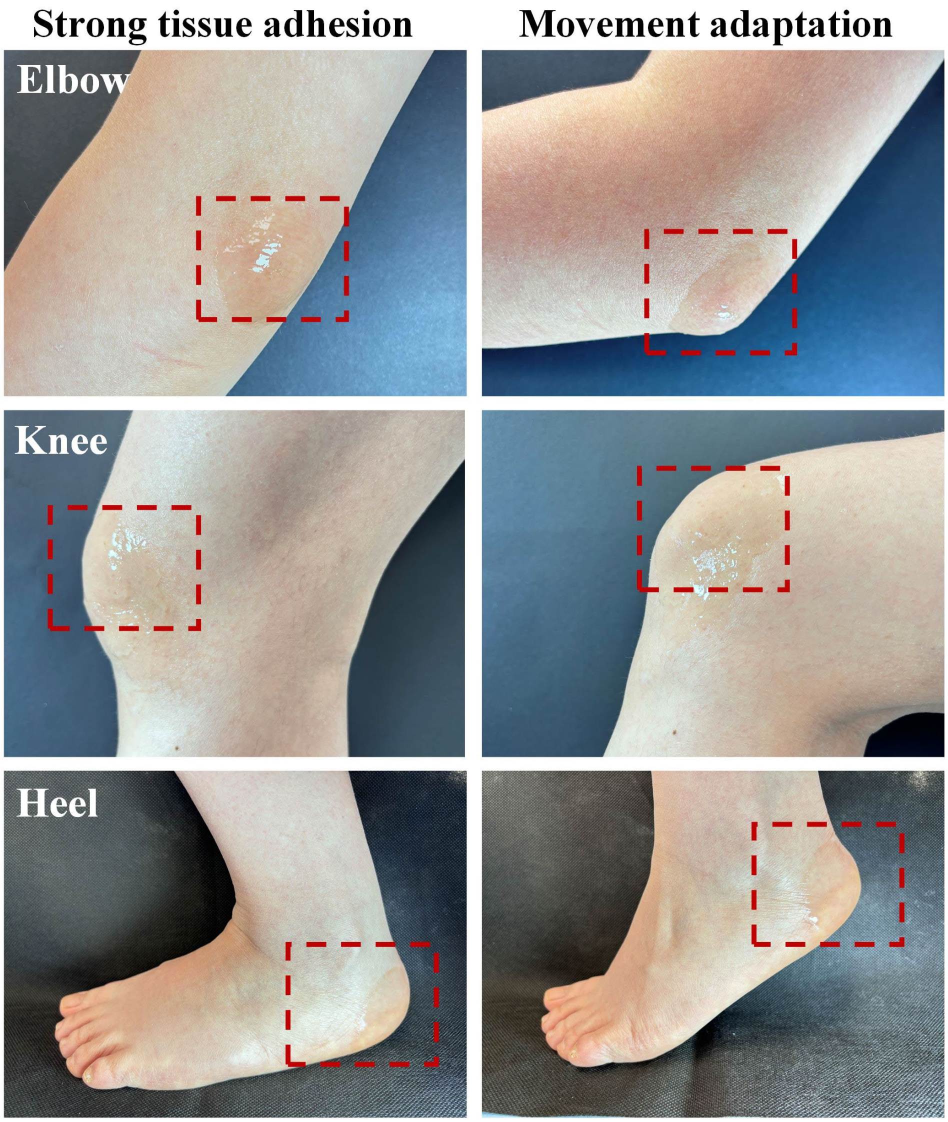

Fig. S10. Adaptability evaluation of our BDMs on different skins with varied mechanical properties and dryness.

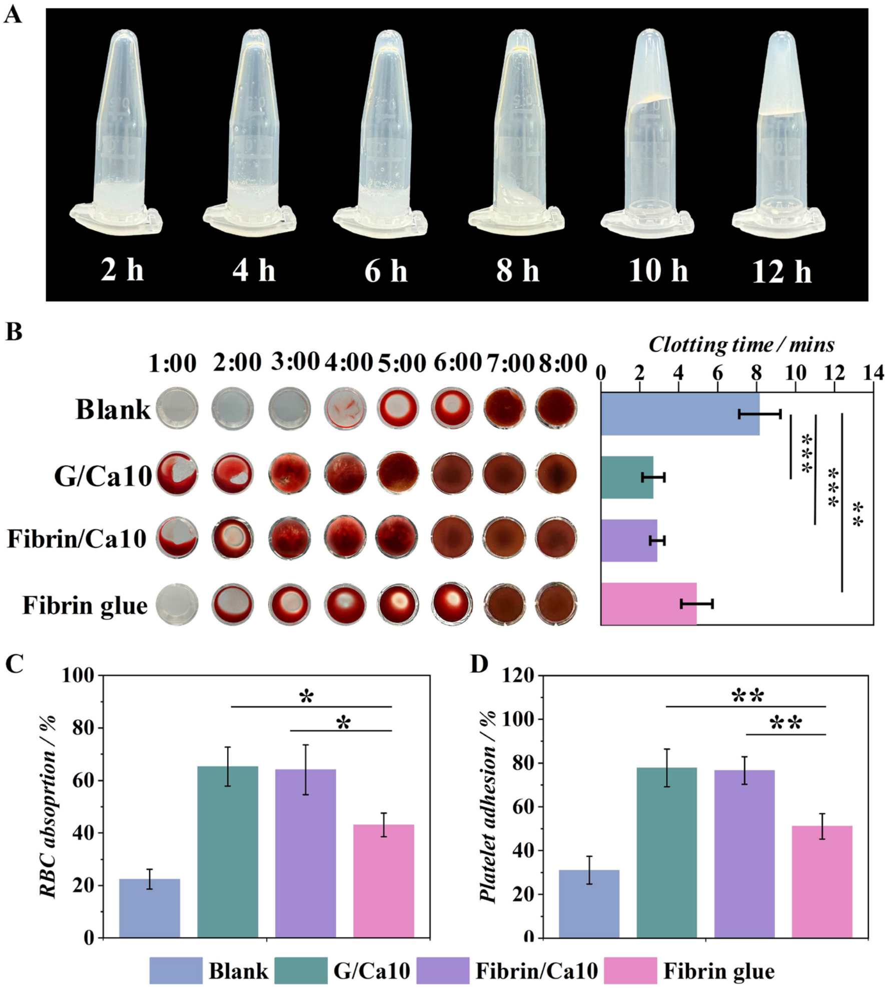

Fig. S11. Hemostasis evaluation of the thrombin-free fibrin glue. (A) Gel formation process of the thrombin-free fibrin glue crosslinked by calcium chloride. (B) Whole blood clotting assay with clotting time quantification of the different samples. Quantification of (C) red blood cells (RBCs) and (D) platelet adhesion of the different samples. Sample size $n = 3$ for all experiments by a one-way ANOVA with a Tukey's post hoc test for multiple comparisons. Data are presented as mean ± SD. *$p < 0.05$, **$p < 0.01$, ***$p < 0.001$ are considered statistically significant.

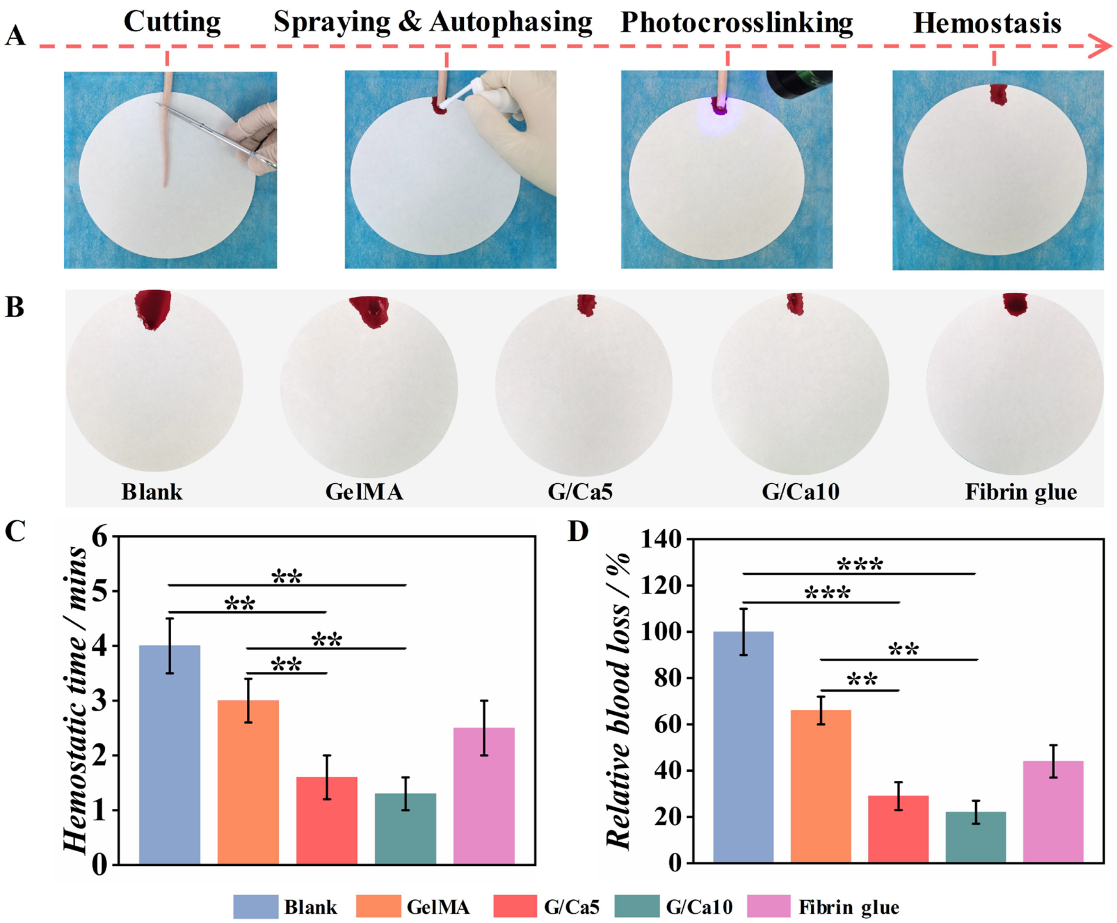

Fig. S12. Evaluation of hemostasis efficacy of the BDMs using rat tail bleeding hemostatic model. (A) Typical experimental procedure and (B) bloodstain images of the rat tail bleeding hemostatic model. Quantification of (C) relative blood loss and (D) hemostasis time for different samples. The G/Ca5, G/Ca10 denoted GelMA with 5%, 10% $CaCl_2$ incorporation, respectively. Sample size $n = 3$ for all experiments by one-way ANOVA with a Tukey's post hoc test for multiple comparisons. Data are presented as mean ± SD. $^{**}p < 0.01$, $^{***}p < 0.001$ are considered statistically significant.

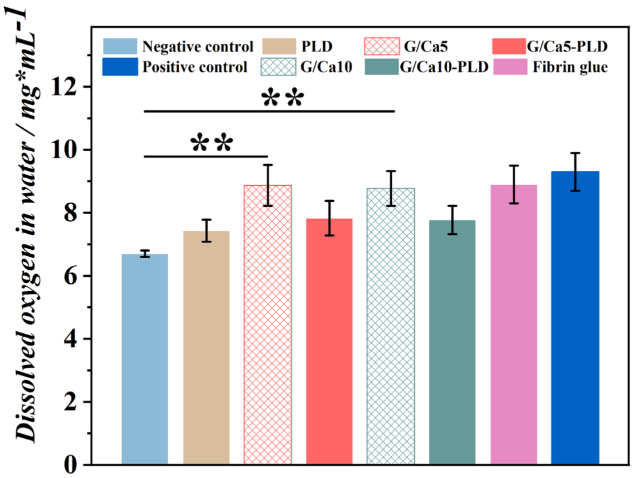

Fig. S13. The oxygen permeability quantification of our BDMs. The open bottle and sealed bottle were used as the positive control and negative control respectively. Sample size n = 3 for all experiments by one-way ANOVA with a Tukey's post hoc test for multiple comparisons. Data are presented as mean ± SD. *p < 0.05, **p < 0.01 are considered statistically significant.

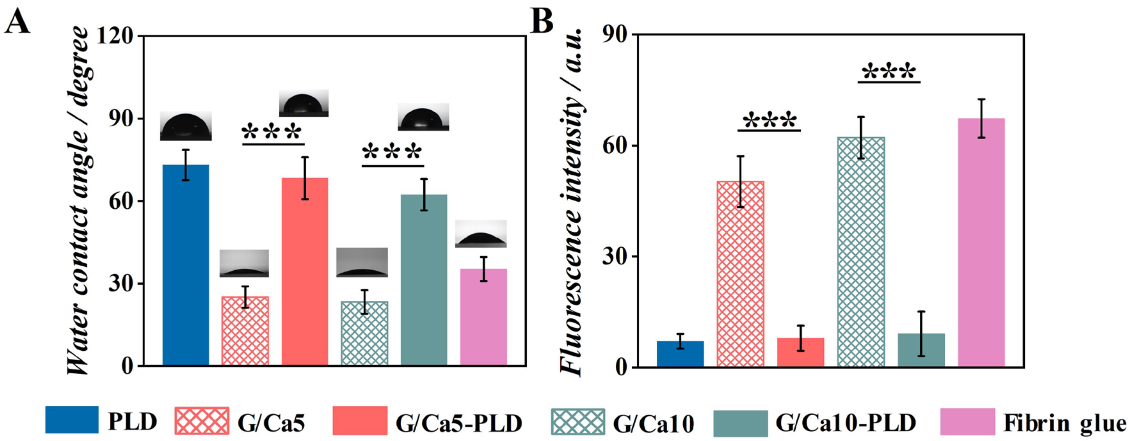

Fig. S14. (A) Water contact angle and (B) fluorescence intensity quantification of the adsorbed BSA-FITC protein on different samples. Sample size $n = 3$ for all experiments by a one-way or two-way ANOVA with a Tukey's post hoc test for multiple comparisons. Data are presented as mean ± SD. *$p < 0.05$, **$p < 0.01$, ***$p < 0.001$ are considered statistically significant.

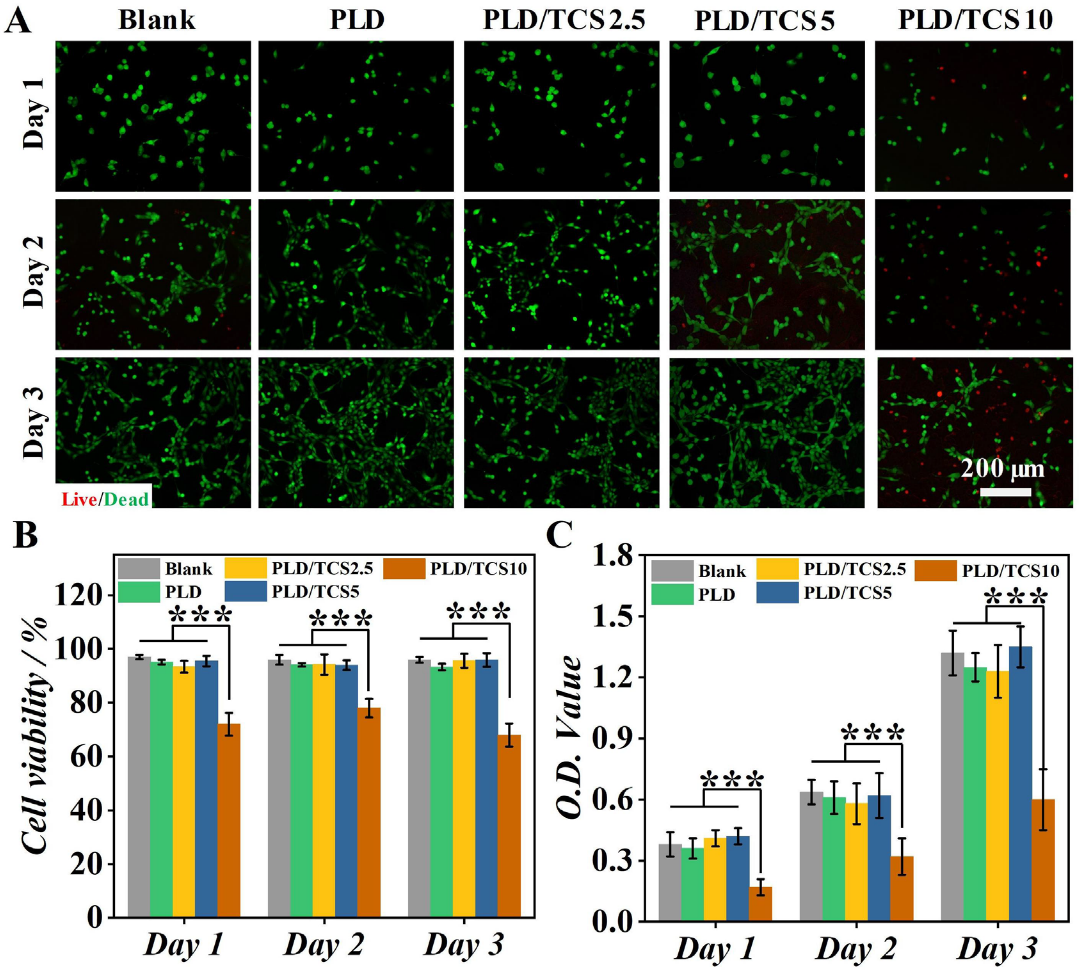

Fig. S15. Biocompatibility evaluation of PLD with different concentration of triclosan (TCS) using the extraction medium of different samples. (A) Live/Dead staining of the NIH/3T3 fibroblasts. Green and red fluorescence indicate the live and dead cells respectively. Quantification of (B) cell viability and (C) cell proliferation on day 1, 2 and 3. Sample size $n = 3$ for all experiments by a two-way ANOVA with a Tukey's post hoc test for multiple comparisons. Data are presented as mean ± SD. $^{*}p < 0.05$, $^{**}p < 0.01$, $^{***}p < 0.001$ are considered statistically significant.

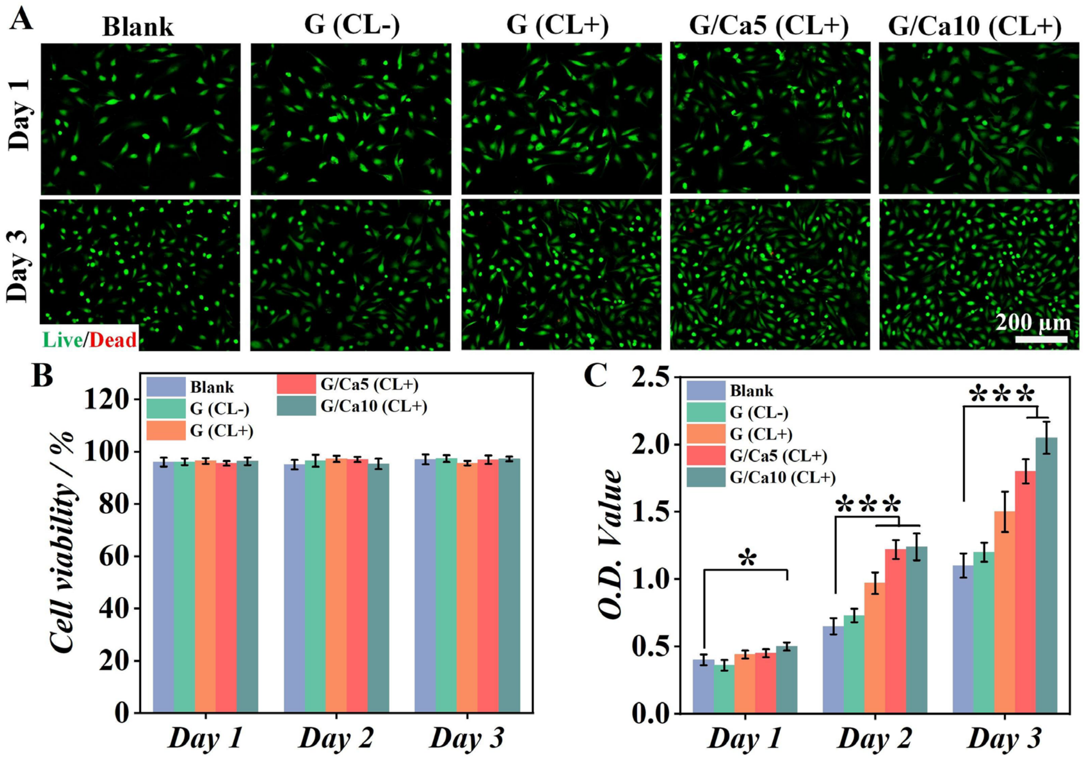

Fig. S16. (A) Live/Dead staining of the HUVECs with different conditioned medium after 1- and 3-day of culture. Green and red fluorescence indicated the live and dead cells respectively. Quantification of (B) cell viability and (C) cell proliferation on day 1, 2 and 3. Sample size $n = 3$ for all experiments by a two-way ANOVA with a Tukey's post hoc test for multiple comparisons. Data are presented as mean ± SD. $*p < 0.05$, $**p < 0.01$, $***p < 0.001$ are considered statistically significant.

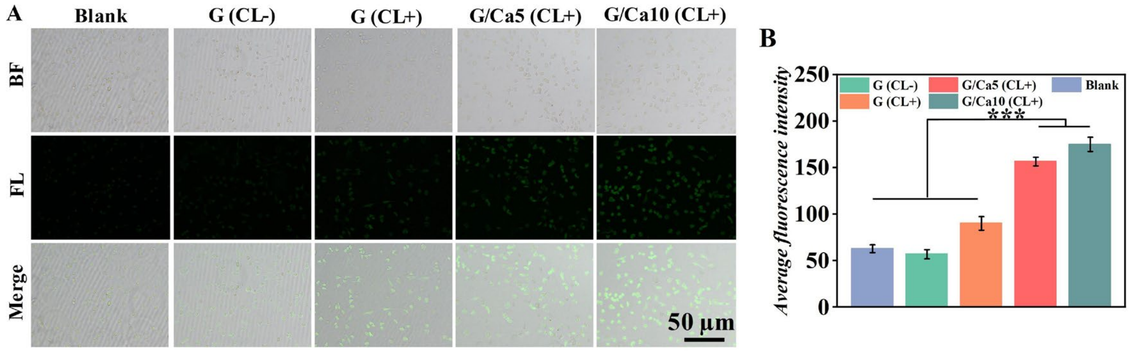

Fig. S17. (A) NO generation in HUVECs detected by the DAF-FM DA fluorescence probe. (B) Quantitative of DAF-FM DA fluorescence signal. Sample size $n = 3$ for all experiments by a one-way ANOVA with a Tukey's post hoc test for multiple comparisons. Data are presented as mean ± SD. $^{**}p < 0.01$ is considered statistically significant.

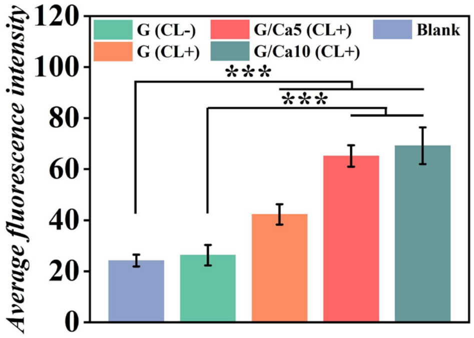

Fig. S18. Quantification of CD31 expression of HUVECs through average fluorescence intensity cultured with different conditioned medium after 3 days of incubation. Sample size $n = 3$ for all experiments by a one-way ANOVA with a Tukey's post hoc test for multiple comparisons. Data are presented as mean ± SD. $^{**}p < 0.01$ is considered statistically significant.

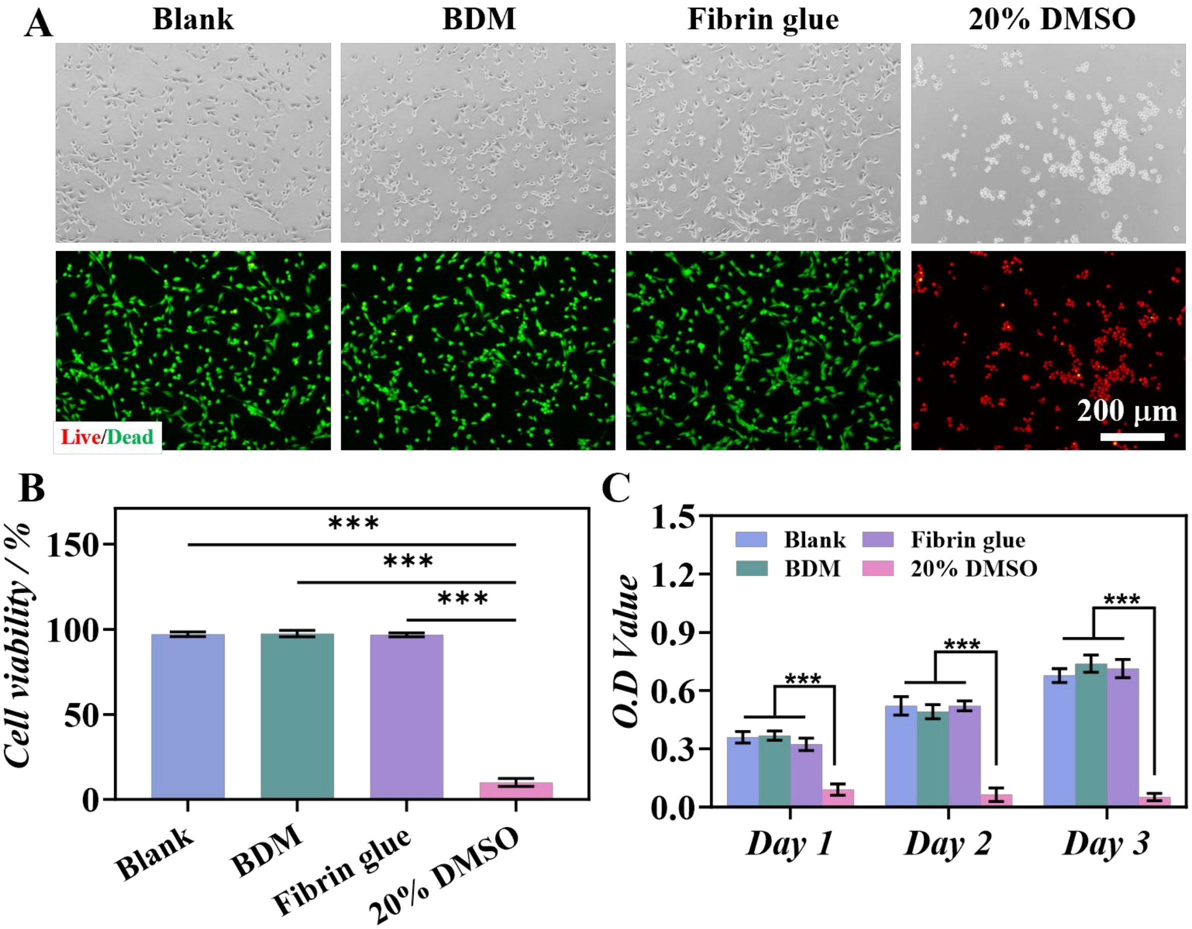

Fig. S19. (A) Bright-field and fluorescent live/dead staining of the NIH/3T3 cells with different conditioned medium after 1 day of culture. Green and red fluorescence indicated the live and dead cells respectively. Quantification of (B) cell viability on day 1 and (C) cell proliferation on day 1, 2 and 3. Sample size $n = 3$ for all experiments by a two-way ANOVA with a Tukey's post hoc test for multiple comparisons. Data are presented as mean ± SD. *$p < 0.05$, **$p < 0.01$, ***$p < 0.001$ are considered statistically significant

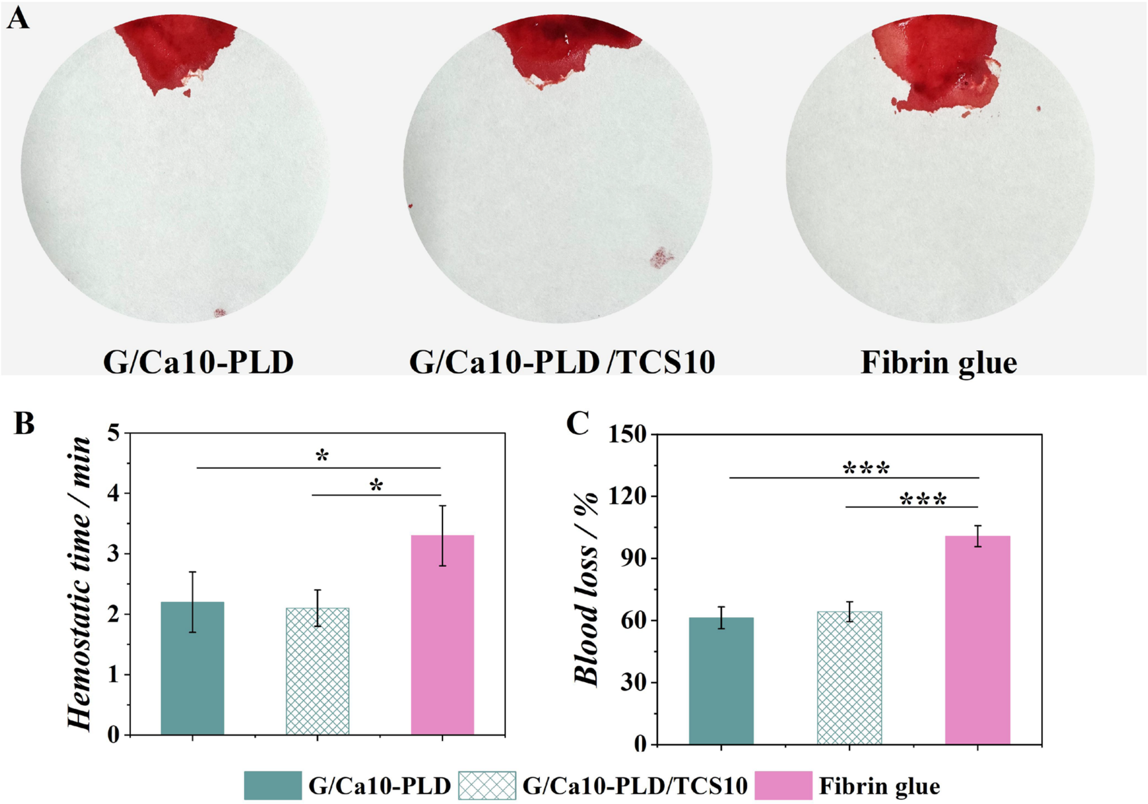

Fig. S20. Hemostasis evaluation of the optimized BDM (G/Ca10 and PLD/TCS5 as the bottom and upper layers respectively) using a rat liver injury model. (A) Bloodstain images with the quantification of (B) hemostasis time and (C) relative blood loss for different samples. Sample size $n = 3$ for all experiments by a one-way ANOVA with a Tukey's post hoc test for multiple comparisons. Data are presented as mean ± SD. *$p < 0.05$, **$p < 0.01$, ***$p < 0.001$ are considered statistically significant.

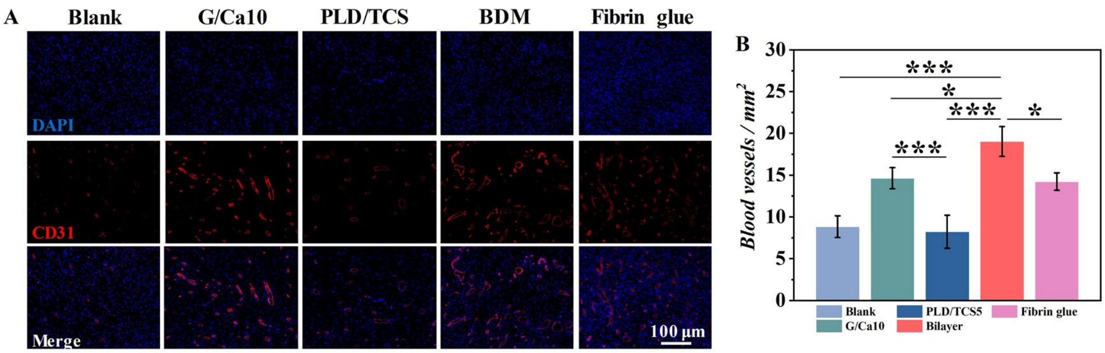

Fig. S21. (A) Immunofluorescence CD31 staining for the wound sections after 14 days. The red and blue color indicated the CD31 and nuclei, respectively. (B) Quantification of the blood vessel density after 14 days. Sample size $n = 3$ for all experiments by a one-way ANOVA with a Tukey's post hoc test for multiple comparisons. Data are presented as mean ± SD. $*p < 0.05$ is considered statistically significant.

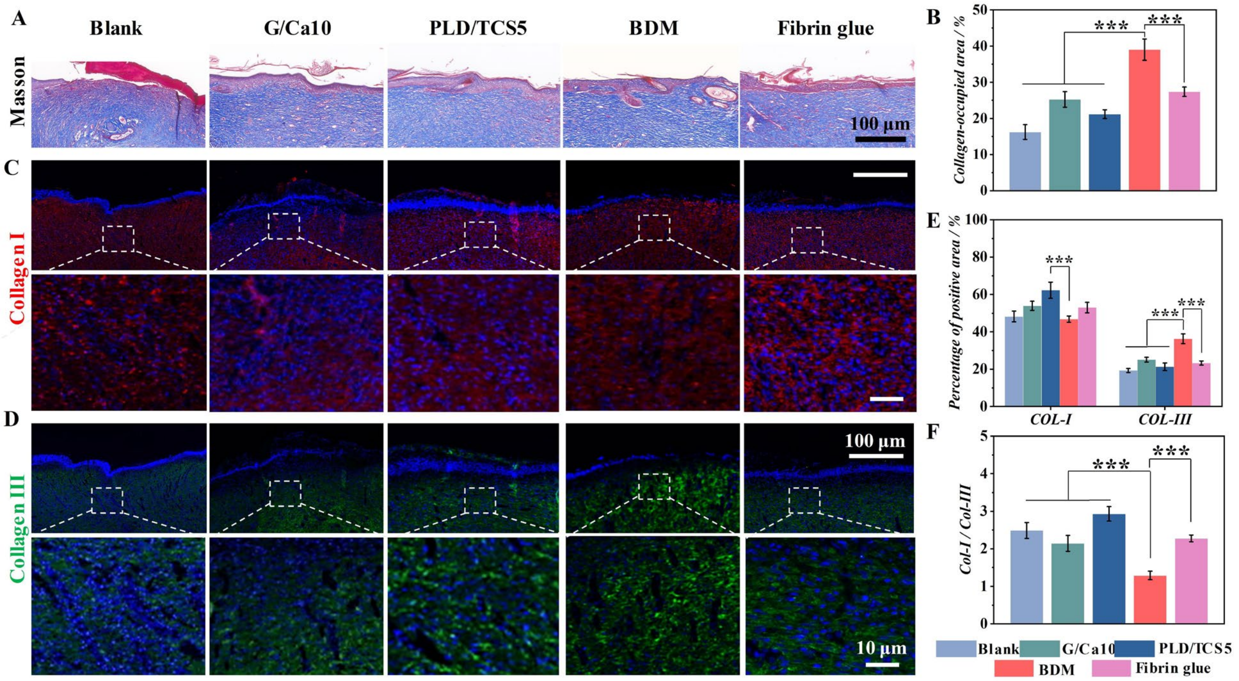

Fig. S22. (A) Masson's trichrome staining with (B) the quantification of collagen occupied area for the wound sections after 14 days. Immunofluorescence staining of (C) type I collagen (Col-I) and (D) type III collagen (Col-III) for the wound sections after 14 days. Quantification of the (E) percentage of positive area and (F) ratio between the Col-I and Col-III. Sample size $n = 3$ for all experiments by a one-way or two-way ANOVA with a Tukey's post hoc test for multiple comparisons. Data are presented as mean ± SD. $^{**}p < 0.01$ and $^{***}p < 0.001$ are considered statistically significant.

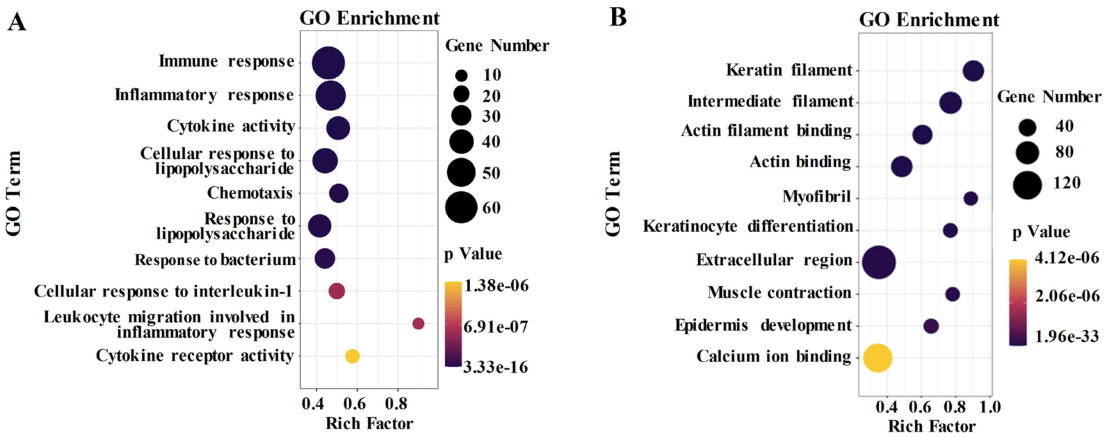

Fig. S23. (A) Downregulated enriched GO terms and (B) upregulated enriched GO terms on day 3 (BDM group versus blank group).

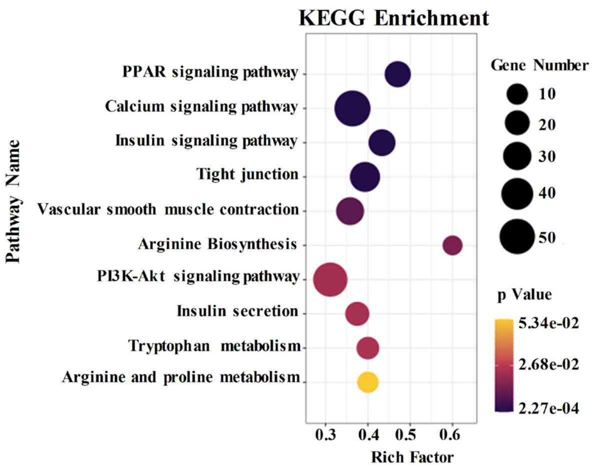

Fig. S24. Upregulated enriched KEGG pathways on day 3 (BDM group versus blank group).

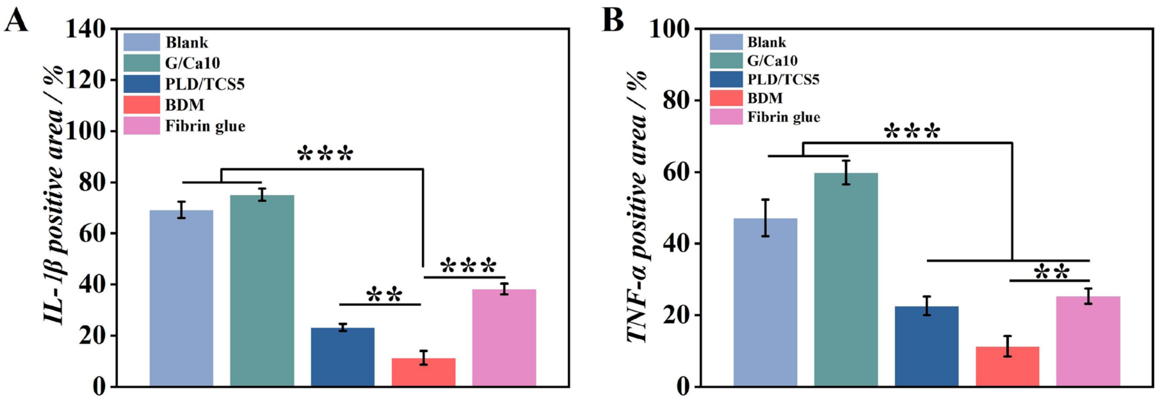

Fig. S25. Quantification of the positive area of immunofluorescence staining of (A) IL-1β and (B) TNF-α of different wound masks on day 3. Sample size $n = 3$ for all experiments by a one-way ANOVA with a Tukey's post hoc test for multiple comparison. Data are presented as mean ± SD. *$p < 0.05$ and ***p <0.001 are considered statistically significant.

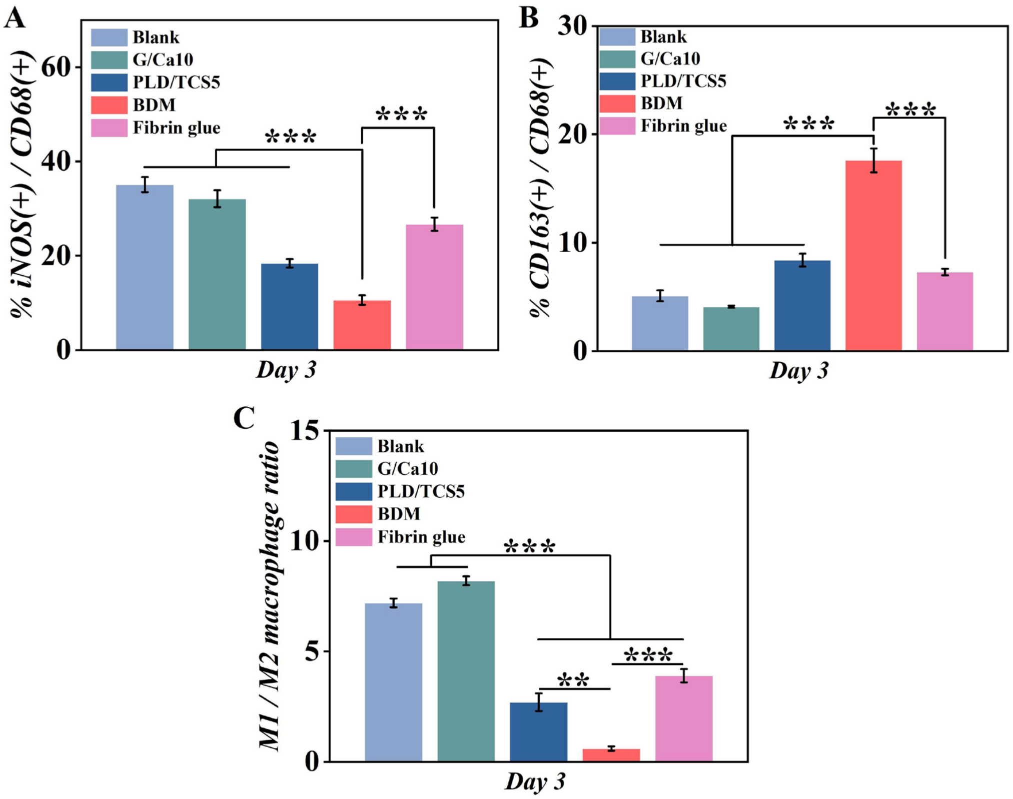

Fig. S26. Quantification of immunofluorescence staining of the (A) iNOS(+)/CD68 (+), (B) CD163(+)/CD68 (+) and (C) the ratio between the M1 and M2 type macrophages of different wound masks on day 3. Sample size $n = 3$ for all experiments by a one-way ANOVA with a Tukey's post hoc test for multiple comparisons. Data are presented as mean ± SD. $^{**}p < 0.01$ and $^{***}p < 0.001$ are considered statistically significant.

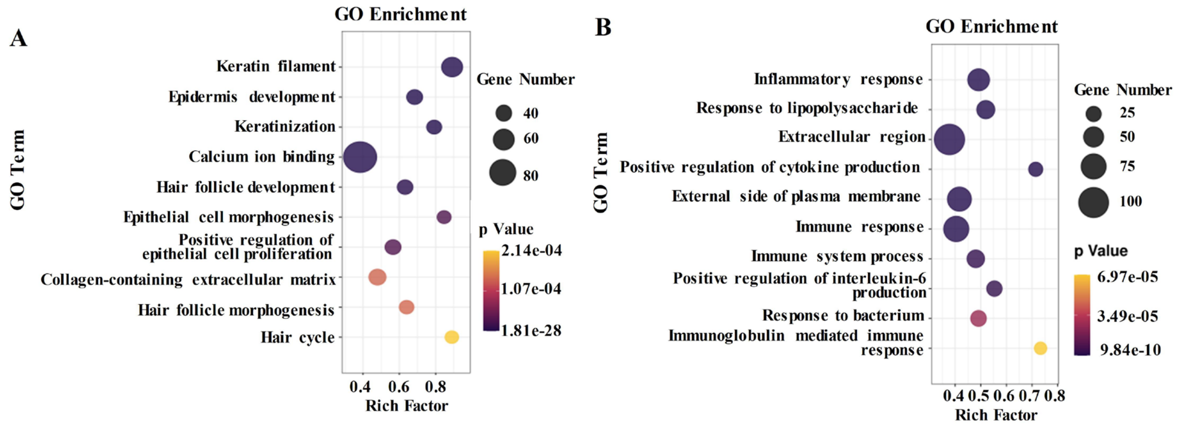

Fig. S27. (A) Upregulated enriched GO terms and (B) downregulated enriched GO terms on day 7 (BDM group versus blank group).

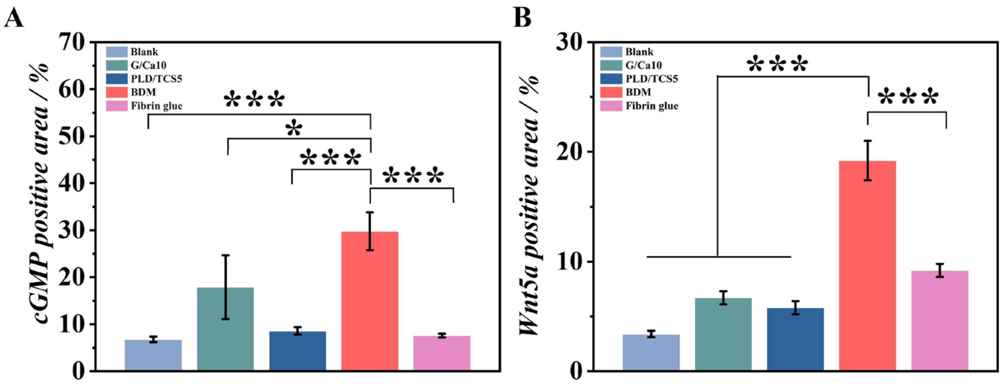

Fig. S28. Quantification of the positive area of immunofluorescence staining of (A) cGMP and (B) Wnt5a of different wound masks on day 7. Sample size $n = 3$ for all experiments by a one-way ANOVA with a Tukey's post hoc test for multiple comparison. Data are presented as mean ± SD. ***p <0.001 is considered statistically significant.

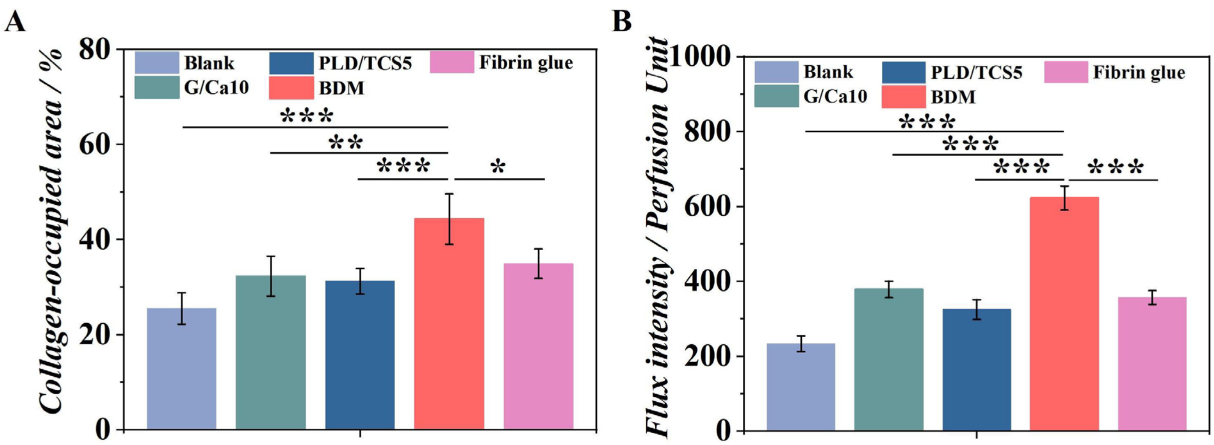

Fig. S29. Quantification of (A) collagen occupied area in Masson's trichrome staining and (B) flux intensity in laser Doppler perfusion imaging. Sample size $n = 5$ for all experiments by a one-way or two-way ANOVA with a Tukey's post hoc test for multiple comparisons. Data are presented as mean ± SD. $*p < 0.05$ and $***p < 0.001$ are considered statistically significant.

Supporting movie legend

Movie S1. The autophasing capacity demonstration of our BDMs with PLD and GelMA with 10 wt.% $CaCl_2$ (G/Ca10) due to spontaneous water/oil separation.
Movie S2. The implementation procedure demonstration of our BDMs for the rapid construction of bi-layered wound mask.
